# ARIH2 Ubiquitination Regulates NUPR1 to Inhibit Ferroptosis in Bladder Cancer

**DOI:** 10.1111/jcmm.71147

**Published:** 2026-04-17

**Authors:** Hannuo Deng, Yuanyuan Mi, Lei Zhang, Yuchen Tan, Guangming He, Zebin Shi, Shenglin Gao, Lifeng Zhang, Li Zuo, Jie Peng

**Affiliations:** ^1^ Department of Urology The Second People's Hospital of Changzhou, Changzhou Medical Center Changzhou China; ^2^ Department of Urology Affiliated Hospital of Jiangnan University Wuxi China; ^3^ Department of Nursing Wuxi Huishan District People's Hospital Wuxi China

**Keywords:** ARIH2, bladder cancer, ferroptosis, macrophage polarization, NUPR1, tumour microenvironment

## Abstract

NUPR1 is a pro‐tumorigenic factor in bladder cancer (BLCA), but the mechanisms governing its protein stability remain poorly defined. Here, we identified ARIH2 as an E3 ubiquitin ligase that interacts with NUPR1 in BLCA cells through immunoprecipitation‐mass spectrometry (IP‐MS), co‐immunoprecipitation (Co‐IP), and immunofluorescence analyses. ARIH2 overexpression reduced NUPR1 abundance and inhibited BLCA cell proliferation and migration while enhancing apoptosis, whereas ARIH2 knockdown increased NUPR1 expression and promoted malignant phenotypes. Mechanistically, ARIH2 depletion prolonged NUPR1 protein stability and reduced its ubiquitination, indicating that ARIH2 negatively regulates NUPR1 through ubiquitin‐mediated degradation. Moreover, NUPR1 overexpression suppressed ferroptosis, as reflected by increased GPX4 and SLC7A11, decreased ACSL4, reduced lipid peroxidation, and diminished Fe^2+^ accumulation, while NUPR1 knockdown exerted the opposite effects. ARIH2 knockdown mimicked the ferroptosis‐resistant phenotype induced by NUPR1 upregulation, supporting that ARIH2 modulates ferroptosis through NUPR1. Bioinformatic and experimental analyses further showed that NUPR1 was associated with immunosuppressive infiltration and promoted M2 macrophage polarization. Together, our findings uncover an ARIH2–NUPR1 regulatory axis that drives BLCA progression by suppressing ferroptosis and favouring an immunosuppressive microenvironment, highlighting this pathway as a potential therapeutic target in BLCA.

**Abbreviations**
BLCA: bladder cancer
CCK‐8: cell counting kit‐8
CHX: cycloheximide
CO_2_
: carbon dioxide
Co‐IP: co‐immunoprecipitation
IP‐MS: immunoprecipitation‐mass spectrometry
MHC: major histocompatibility complex
PTM: posttranslational modification
RT‐qPCR: real‐time fluorescence quantitative PCR
TAMs: tumour‐associated macrophages
TME: tumour microenvironment
WB: western blotting

## 1 Introduction

Bladder cancer (BLCA) is a common malignancy with frequent recurrence and progression, posing a substantial clinical burden worldwide. Despite advances in surgical management and systemic therapies for advanced disease, durable therapeutic responses remain limited for a considerable proportion of patients. Therefore, identifying key molecular drivers and actionable pathways is essential to improve BLCA outcomes [1, 2, 3, 4].

*NUPR1* is a small nuclear protein involved in many biological processes, such as transcriptional regulation, cell cycle regulation, apoptosis and autophagy [5, 6]. NUPR1 has been found to play important roles in a variety of cancers. Our previous studies confirmed that NUPR1 plays a role in promoting the proliferation, migration and invasion of bladder cancer cells [7, 8], but the specific mechanism is not clear. Recent studies have further identified *MYH11* as an upstream transcriptional regulator of *NUPR1*, which activates the PI3K/AKT pathway to promote bladder cancer progression. This suggests that NUPR1 may function within a broader regulatory network involving both transcriptional and post‐translational mechanisms [9, 10]. Ubiquitination is a posttranslational modification (PTM) process in which single or multiple ubiquitins are covalently bound to a target protein, which plays a key role in protein localization, metabolism and degradation. This process is involved in the regulation of various signal transduction processes related to immunity, inflammation and cancer [11, 12]. The ubiquitination process proceeds through the sequential action of three enzymes, including an E1‐activating enzyme, an E2‐conjugating enzyme, and an E3 ubiquitin ligase [13]. The *ARIH2* gene is a member of the Ariadne subfamily and encodes a protein with E3 ubiquitin ligase activity. Previous studies have reported that ARIH2 is involved in p53‐associated stress responses and facilitates Nutlin‐3a–induced p53 activation, thereby enhancing the growth‐inhibitory and cytotoxic effects of Nutlin‐3a in human cancer cells. Conversely, ARIH2 silencing attenuates the anti‐tumour efficacy of Nutlin‐3a [14]. Therefore, ARIH2 may play an antagonistic role in tumorigenesis.

## 2 Materials and Methods

### 2.1 Cell Lines

The human BLCA cell line T24 was purchased from the Chinese Academy of Sciences (Shanghai, China). The T24 cell line was derived from a female patient with grade III BLCA. The cells were cultured in Roswell Park Memorial Institute‐1640 medium (Gibco, USA), which included 10% fetal bovine serum, 100 U/mL penicillin, and streptomycin, in a 5% carbon dioxide (CO_2_) incubator at 37°C. The medium was changed every 3 days for the cell lines. The cells were harvested when they reached approximately 80% confluence.

### 2.2 Western Blot (WB)

Protein expression was determined by Western blotting as follows: cells were lysed with Beyotime Biotechnology IP lysis buffer (P0013, China). Protein concentrations were determined via a BCA protein assay kit (Beyotime, China). The absorbance was measured at 562 nm via a microplate spectrophotometer (Fisher Scientific, USA). Proteins (10 μg) were separated on a 10% SDS–polyacrylamide gel and then transferred to a nitrocellulose membrane at 200 mA for 85 min. Membranes were incubated with primary antibodies overnight at 4°C. Finally, the protein bands were visualized with an enhanced chemiluminescence western blotting substrate kit (Abcam, UK). Relative protein expression was analysed via Quantity One software, with GAPDH used as an internal reference.

### 2.3 Real‐Time Fluorescence Quantitative PCR (RT‐qPCR)

Total RNA was isolated from tissues and cells with the help of TRIzol reagent (Sigma, USA). The concentration and purity of the RNA samples were measured via a Nanodrop 2000 (Thermo Fisher Scientific, USA). Total RNA was reverse transcribed into cDNA via the PrimeScript RT Kit (Vazyme, China). RT‐qPCR was subsequently performed via TB Green Premix Ex Taq II (Vazyme, China) and an ABI Prism 7500 Sequence Detection System (Applied Biosystems, USA).

### 2.4 Immunoprecipitation and Mass Spectrometry Analysis

T24 cells were seeded in 6‐cm dishes and transfected with a vector plasmid or a Flag‐tagged NUPR1 plasmid. The cells were lysed as described above and immunoprecipitated with anti‐igg or anti‐Flag antibodies 48 h later. The successful immunoprecipitation of NUPR1 was verified via silver staining and Western blotting. The immunoprecipitated proteins were then subjected to liquid chromatography and tandem mass spectrometry for proteomic analysis (Novogene, China).

### 2.5 Co‐Immunoprecipitation (Co‐IP)

Total protein was extracted after lysis of T24 cells, and 1.0 mg of protein was removed and incubated with the reference antibody at 4°C overnight in an inverted position. The protein‐antibody complex was incubated with 200 μL of microbeads for 1 h at 4°C upside down. The protein–antibody–bead complexes were washed twice with IP lysis buffer and then subjected to SDS–PAGE. After that, the same procedures described above for Western blotting were performed to detect the expression of relevant proteins.

### 2.6 Cell Proliferation Assay

A total of 2 × 10^3^ cells were seeded per well in each well of a 96‐well plate. Cell proliferation was measured every 24 h via a Cell Counting Kit‐8 (CCK‐8) (Sigma, USA). In brief, a solution containing 10 μL of CCK‐8 reagent was added to each well. After 2 h, the absorbance was measured at 450 nm via a spectrometer. Cell viability was measured after 24, 48, 72, 96, and 120 h on the basis of the relative optical density. All the experiments were repeated three times.

### 2.7 Transwell Assay

Cell migration was analyzed via a Transwell migration assay on the basis of previously published data on cell growth. First, cell migration and invasion were measured with a 24‐well Transwell chamber (Corning Life Sciences, USA) with an 8 μm‐well membrane. Bladder cancer cells (20,000) were incubated in serum‐free medium for 24 h and then seeded into the upper chamber of the Transwell insert, and medium containing 10% FBS was added to the lower chamber as a chemoattractant. The chambers were incubated in a cell incubator at 37°C for 48 h, carefully washed twice with PBS, and then fixed at low temperature in 100% methanol for 15 min. Then, the cells were stained with 0.05% crystal violet at room temperature, and five randomly selected areas were analyzed. All the experiments were performed in triplicate.

### 2.8 Protein Stability Assay

To assess the effect of ARIH2 on NUPR1 protein stability, T24 cells were treated with cycloheximide (CHX), a protein synthesis inhibitor. The cells were harvested at 0, 4, 8, and 12 h after CHX treatment for protein extraction. The protein level of NUPR1 was detected by Western blotting.

### 2.9 Ubiquitination Level Detection

After T24 cells were treated with the proteasome inhibitor MG‐132 for 24 h, the proteins in the cells were extracted. A total of 20 μg of protein was removed, and the level of NUPR1 protein was detected via Western blotting. Another 1.0 mg of protein was removed and incubated with the reference antibody overnight at 4°C, followed by upside‐down incubation with 200 μL of microbeads at 4°C for 1 h. Finally, the ubiquitin level of the NUPR1 protein was detected via Co‐IP with anti‐ubiquitin antibodies.

### 2.10 C11‐BODIPY 581/591 Staining for Lipid Peroxidation

Lipid peroxidation was evaluated using the oxidation‐sensitive fluorescent probe BODIPY 581/591 C11 (HY‐D1301, MedChemExpress). Briefly, bladder cancer cells were seeded in culture plates and allowed to adhere overnight. After the indicated treatments, cells were washed twice with PBS and incubated with BODIPY 581/591 C11 working solution (2–10 μM, diluted in serum‐free medium) for 30 min at room temperature in the dark. Subsequently, cells were washed gently with PBS to remove excess probe, and fresh serum‐free medium was added. Fluorescence images were captured immediately using a fluorescence microscope. The oxidation‐dependent fluorescence shift of BODIPY 581/591 C11 was used to assess intracellular lipid peroxidation levels.

### 2.11 Intracellular Fe^2+^ Measurement

Intracellular iron levels were quantified using an iron assay kit based on the ferrozine microplate method (Beijing Leagene Biotechnology). Cell lysates were prepared by homogenization, followed by low‐speed centrifugation to collect the supernatant. For measurement, samples (75 μL) were added to a 96‐well plate, mixed with Fe Assay Buffer (200 μL), and the baseline absorbance was recorded at 562 nm. Subsequently, ferrozine chromogenic solution (8.4 μL) was added to each well, and the mixture was incubated at room temperature for 15 min (or at 37°C for 10 min) before reading absorbance at 562 nm. Iron content was calculated according to the manufacturer's formula, and assays were performed with blank and standard controls.

### 2.12 Flow Cytometry

Flow cytometry detection of apoptosis: When T24 cells reached 85% coverage, they were harvested after trypsin digestion. The cells were washed once with precooled D‐Hanks and 1 × binding buffer. The cells were resuspended in 200 μL of 1× binding buffer and then incubated with 10 μL of annexin V‐APC (Apoptosis Kit, eBioscience, USA) at room temperature in the dark for 15 min. Finally, the degree of apoptosis was detected via flow cytometry (Millipore, USA). Flow cytometry cell cycle assay: When T24 cells coverage exceeded 70%, the cells were harvested after trypsin digestion. After one wash with precooled PBS, the cells were immobilized in precooled 70% ethanol at 4°C for 1 h. After being washed with PBS, the cells were suspended in the prepared staining solution, and the cell cycle distribution was detected via flow cytometry.

### 2.13 Statistical Analysis

All quantitative data are presented as mean ± SD. Error bars represent biological replicates unless otherwise specified. Statistical analyses were performed using R (version 4.3.1) and GraphPad Prism (version 9.3.0). Comparisons between two groups were performed using two‐tailed Student's *t*‐test. Comparisons among three or more groups were conducted using one‐way ANOVA, followed by Tukey's post hoc test. Spearman's correlation coefficient was used for correlation analyses. *p* value < 0.05 was considered statistically significant and statistical significance was denoted as *p < 0.05, **p < 0.01, and ***p < 0.001. Public transcriptome and immune infiltration data of bladder cancer and pan‐cancer cohorts were downloaded from TCGA and TIMER2.0 [15]. Correlation between *NUPR1* expression and immune cell subsets was calculated using Spearman's correlation coefficient, and the results were visualized as BLCA‐specific lollipop plots and pan‐cancer heatmaps using the R ggplot2 package.

### 2.14 Schematic Diagram Construction

The schematic diagram illustrating the ARIH2–NUPR1 axis was created using the online tool BioGDP [16].

## 3 Results

### 3.1 IP–MS Screens for Ubiquitination‐Associated Proteins That Interact With NUPR1

**FIGURE 1:**
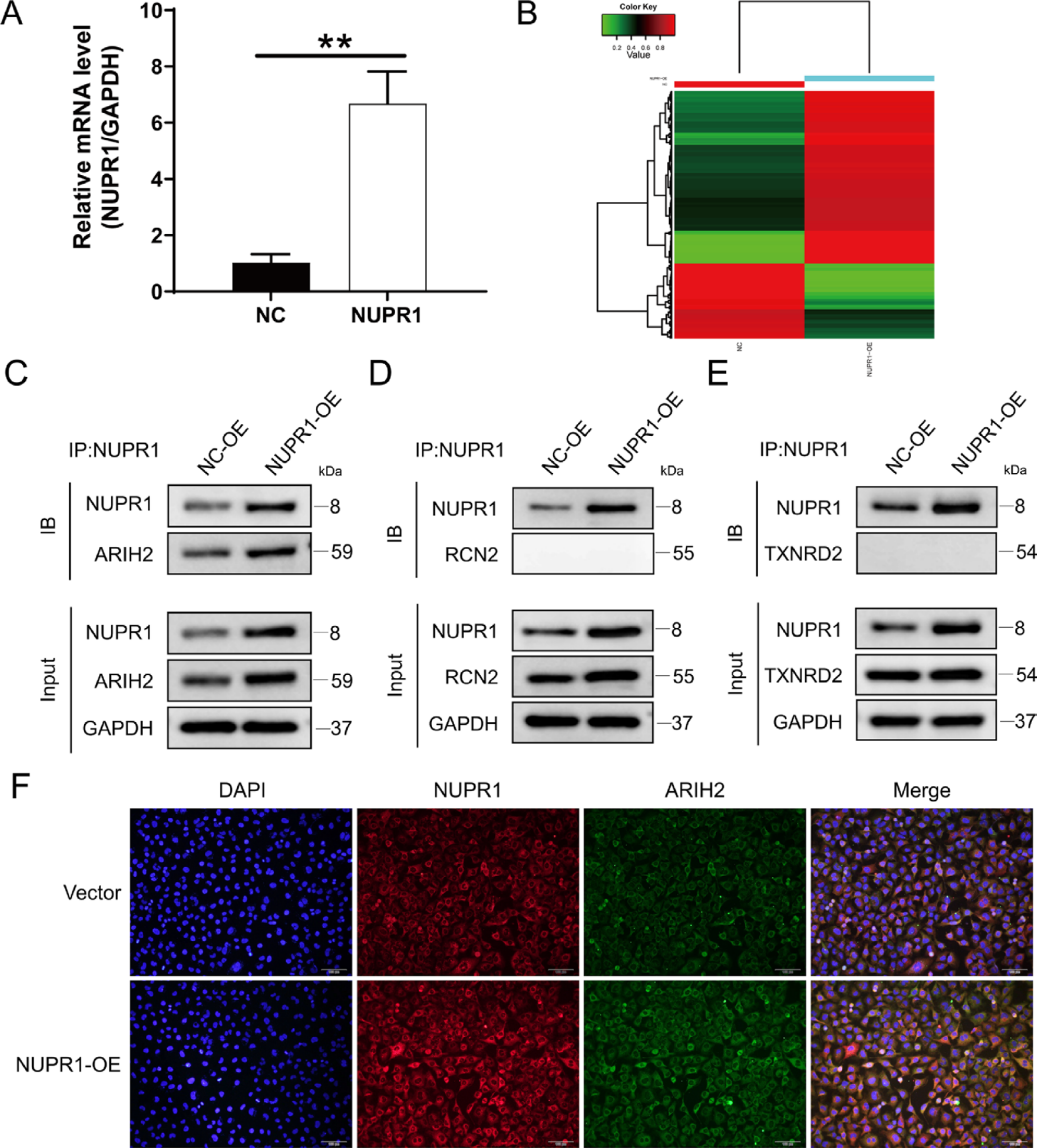
ARIH2 interacts with NUPR1 in bladder cancer cells. (A) NUPR1 overexpression was confirmed via PCR analysis. (B) Identification of NUPR1‐interacting proteins through IP–MS screening. (C–E) Co‐IP was employed to validate the interaction between NUPR1 and the ubiquitin‐related proteins ARIH2, RCN2, and TXNRD2. (F) Immunofluorescence staining provided evidence for the colocalization of NUPR1 and ARIH2 in the cytoplasm.

To further explore the molecular mechanism by which NUPR1 regulates bladder cancer, we first overexpressed NUPR1 in the bladder cancer T24 cell line (Figure 1A) and performed IP‐MS with anti‐IgG or anti‐NUPR1. Bioinformatics analysis was subsequently used to identify proteins that bind to NUPR1, including three ubiquitination‐related proteins, RCN2, TXNRD2, and ARIH2 (Figure 1B). We then used Co‐IP to validate that ubiquitination‐associated proteins interact with NUPR1 in T24 cells (Figure 1C–E). The IP results revealed that the E3 ubiquitin ligase ARIH2 interacts with NUPR1. Moreover, the immunofluorescence results also revealed that the colocalization of NUPR1 and ARIH2 increased after overexpression (Figure 1F). These results suggest that ARIH2 interacts with NUPR1. These results established the direct interaction basis between NUPR1 and ARIH2, providing an experimental basis for subsequent functional studies.

### 3.2 ARIH2 Regulates the Function of Bladder Cancer Cells by Affecting the Protein Level of NUPR1

**FIGURE 2:**
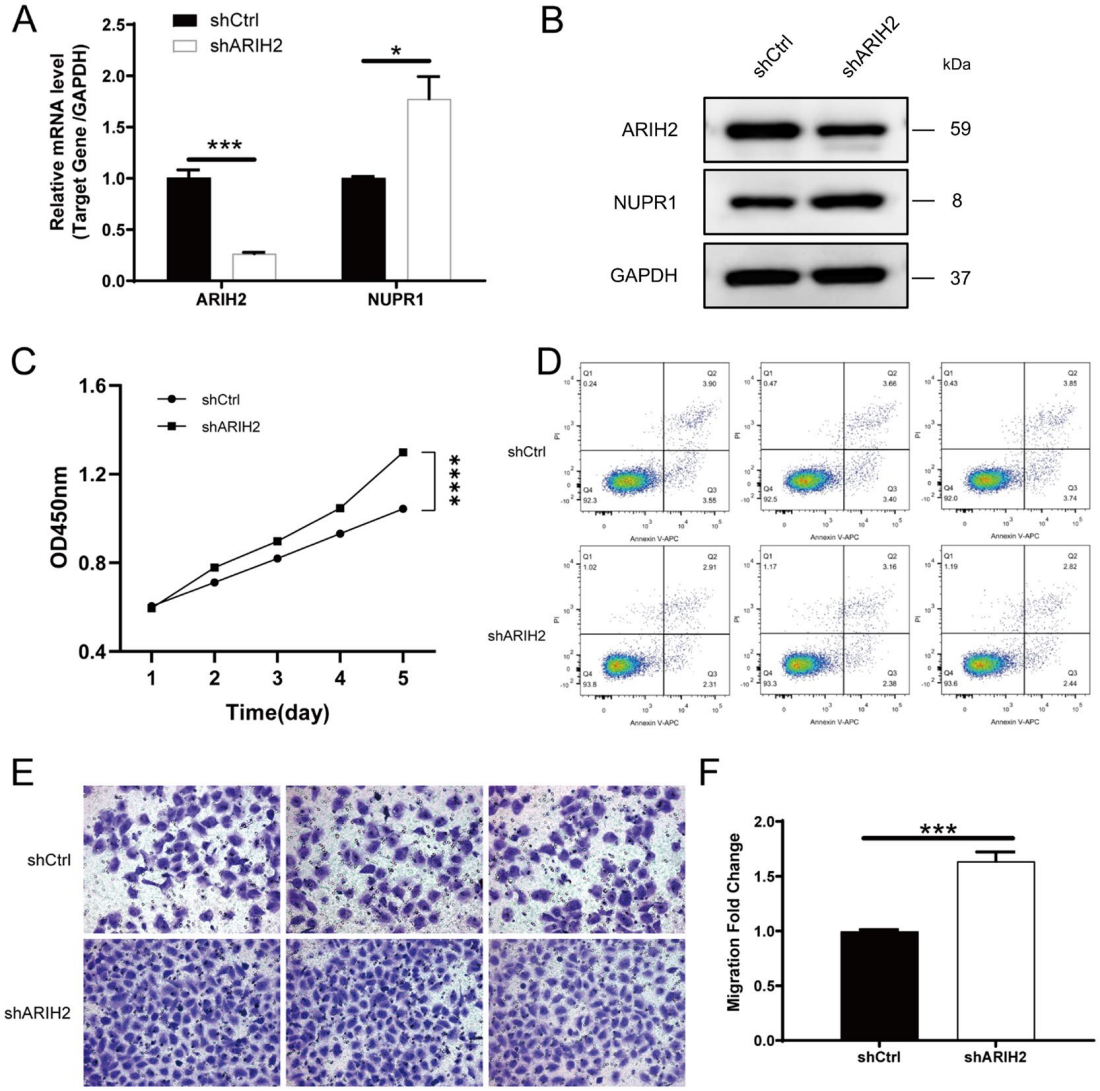
ARIH2 knockdown increases NUPR1 expression and promotes malignant phenotypes of bladder cancer cells. (A) Following shARIH2, *NUPR1* transcript levels in bladder cancer cell lines were determined via PCR analysis. (B) Western blotting was used to detect the NUPR1 protein level in shARIH2 bladder cancer cell lines. (C) Proliferation ability of shARIH2 bladder cancer cells was assessed via the CCK‐8 assay. (D) Apoptosis levels of shARIH2 bladder cancer cells were evaluated via flow cytometry. (E, F) Migration ability of shARIH2 bladder cancer cells was verified via the Transwell assay. Data are presented as mean ± SD from three independent experiments. Statistical significance was determined using two‐tailed Student's *t*‐test. Significance levels: *p < 0.05, **p < 0.01, ***p < 0.001.

**FIGURE 3:**
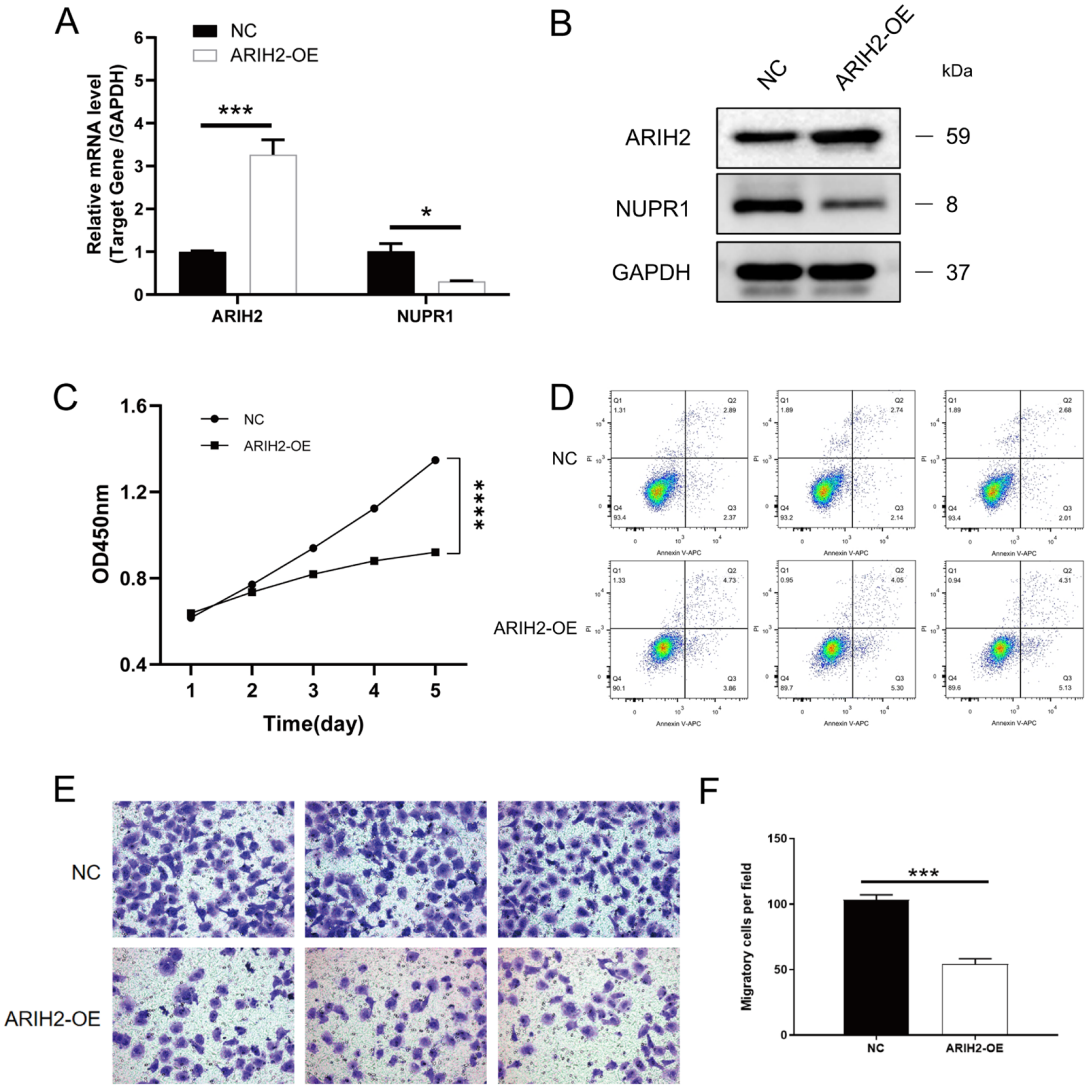
ARIH2 overexpression reduces NUPR1 expression and suppresses malignant phenotypes of bladder cancer cells. (A) Following the overexpression of ARIH2, PCR was used to detect the transcription level of *NUPR1* in a bladder cancer cell line. (B) Protein expression level of NUPR1 in a bladder cancer cell line overexpressing ARIH2 was tested via WB. (C) Proliferation ability of bladder cancer cells overexpressing ARIH2 was assessed via a CCK‐8 assay. (D) Flow cytometry was used to measure the level of apoptosis in bladder cancer cells overexpressing ARIH2. (E, F) Transwell assays were conducted to validate the migration ability of bladder cancer cells overexpressing ARIH2. Data are presented as mean ± SD from three independent experiments. Statistical significance was determined using two‐tailed Student's *t*‐test. Significance levels: *p < 0.05, **p < 0.01, ***p < 0.001.

**FIGURE 4:**
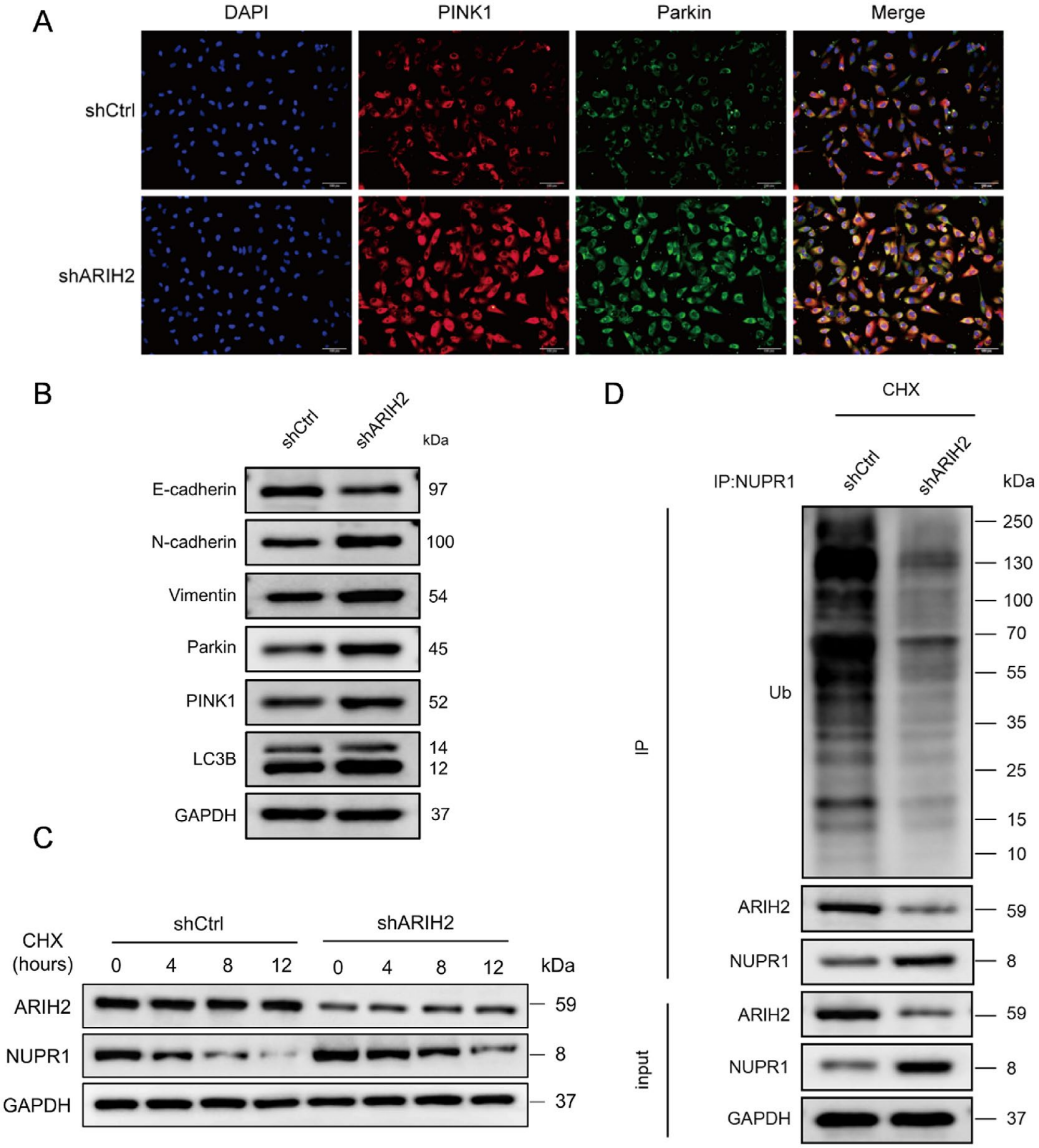
ARIH2 knockdown enhances EMT‐ and mitophagy‐related changes and stabilizes NUPR1. (A) Immunofluorescence staining of PINK1 and Parkin in control and ARIH2‐knockdown cells. (B) Western blot analysis of EMT‐ and autophagy/mitophagy‐related proteins (E‐cadherin, N‐cadherin, Vimentin, PINK1, Parkin, LC3B) in shCtrl and shARIH2 cells. (C) CHX chase assay showing time‐dependent changes of NUPR1 stability after ARIH2 knockdown. (D) Co‐IP of NUPR1, followed by immunoblotting for ubiquitin in shCtrl and shARIH2 cells after CHX treatment to assess the ubiquitination level of NUPR1.

To verify whether ARIH2 can interact with NUPR1 to regulate its effect on the function of bladder cancer cells, we constructed bladder cancer cell lines with ARIH2 knockdown and ARIH2 overexpression and conducted corresponding functional experiments. The results revealed that after ARIH2 knockdown, the expression of NUPR1 increased (Figure 2A,B), the proliferation and migration of tumour cells increased (Figure 2C,E,F), and the number of apoptotic cells significantly decreased (Figure 2D). Similarly, after ARIH2 overexpression, the expression of NUPR1 decreased (Figure 3A,B), the proliferation and migration ability of tumour cells decreased (Figure 3C,E,F), and the number of apoptotic cells increased (Figure 3D). Immunofluorescence results also revealed increased expression of PINK1 and Parkin (Figure 4A). Subsequent WB results revealed that E‐cadherin expression decreased, N‐cadherin and vimentin expression increased, and the expression of the autophagy‐related proteins PINK1, Parkin and LC‐3I/II increased in cells with ARIH2 knockdown (Figure 4B). These results demonstrate that ARIH2 regulates the expression level of NUPR1, thereby affecting the proliferation, migration, apoptosis and autophagy of bladder cancer cells.

### 3.3 ARIH2 Knockdown Increased NUPR1 Protein Levels by Inhibiting Ubiquitination

The cells were treated with the protein synthesis inhibitor CHX at different time points (0, 4, 8, and 12 h), and samples were collected for Western blot analysis to determine the ubiquitination process of NUPR1 by ARIH2. The WB results revealed that ARIH2 knockdown promoted the stability of NUPR1 and delayed its degradation (Figure 4C). Moreover, the level of ubiquitinated NUPR1 in the ARIH2‐knockdown control group and the ARIH2‐knockdown group was detected via WB after anti‐NUPR1 antibody immunoprecipitation, and the results revealed that ARIH2 knockdown significantly reduced the level of ubiquitinated NUPR1 (Figure 4D). These results suggest that ARIH2 knockdown stabilizes NUPR1 by reducing the level of ubiquitination.

### 3.4 ARIH2 Regulates Ferroptosis via NUPR1

**FIGURE 5:**
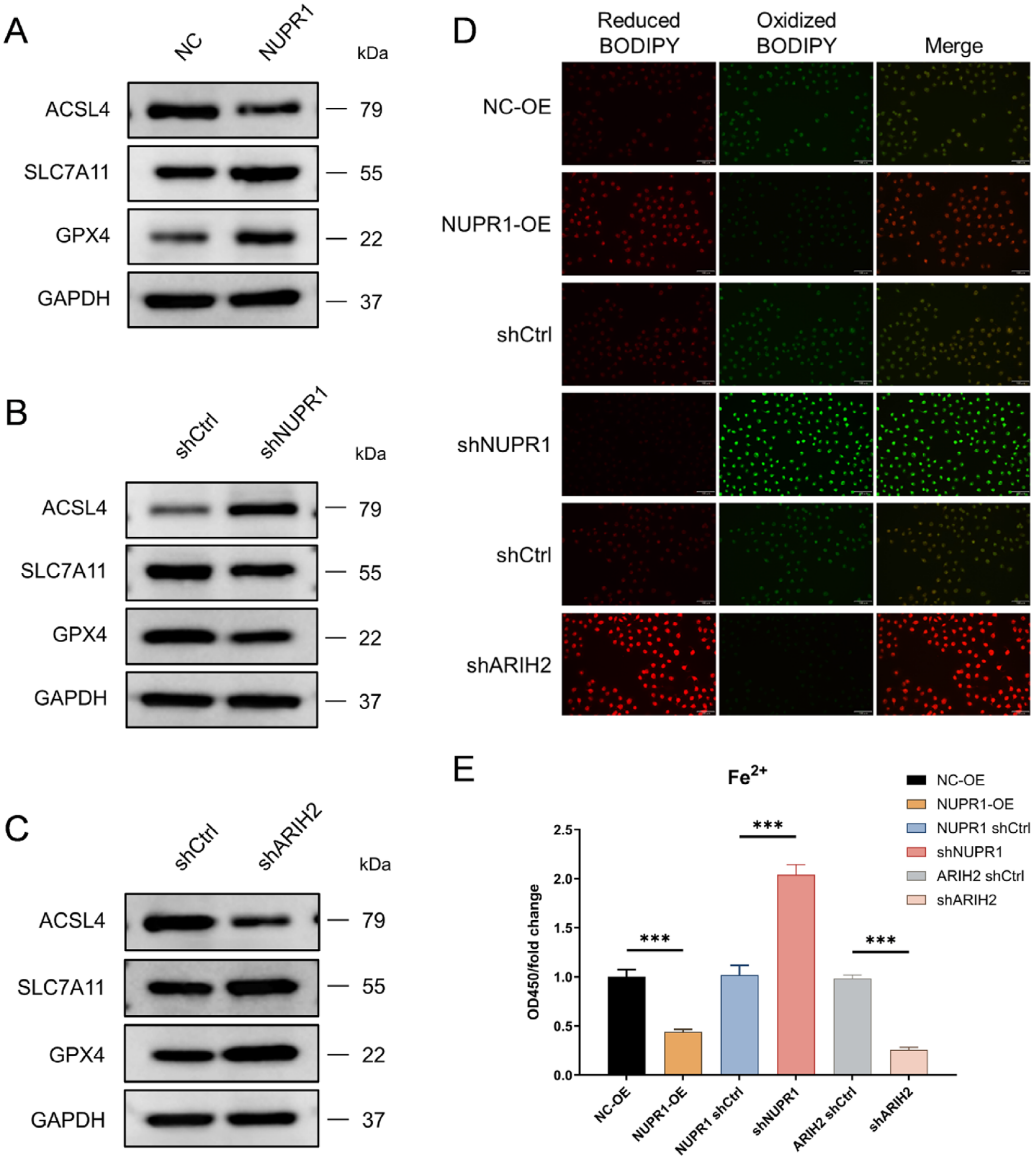
ARIH2–NUPR1 axis regulates ferroptosis in bladder cancer cells. (B) Western blot analysis of ACSL4, SLC7A11 and GPX4 after shNUPR1. (C) Western blot analysis of ACSL4, SLC7A11 and GPX4 following ARIH2 knockdown. (D) C11‐BODIPY 581/591 fluorescence staining was used to evaluate intracellular lipid peroxidation levels. (E) Intracellular ferrous iron (Fe^2+^) levels were quantified using a commercial iron assay kit. Data are presented as mean ± SD. Statistical significance was determined using one‐way ANOVA, followed by Tukey's post hoc test. Significance levels: *p < 0.05, **p < 0.01, ***p < 0.001.

Overexpression of NUPR1 suppressed ferroptosis, as indicated by increased expression of GPX4 and SLC7A11 and reduced ACSL4. In contrast, knockdown of NUPR1 promoted ferroptosis. Similarly, knockdown of ARIH2 produced effects resembling NUPR1 overexpression, supporting that ARIH2 regulates ferroptosis through modulation of NUPR1 (Figure 5A–C). The uncropped full‐length western blot images are provided in Figure S1. To further validate whether ARIH2 regulates ferroptosis through NUPR1, we assessed ferroptosis‐associated lipid peroxidation and intracellular ferrous iron (Fe^2+^) levels. C11‐BODIPY 581/591 staining showed that NUPR1 overexpression reduced lipid peroxidation, whereas NUPR1 knockdown markedly increased C11‐BODIPY oxidation. Consistently, Fe^2+^ quantification revealed concordant changes in intracellular iron accumulation among these groups. Notably, ARIH2 knockdown phenocopied the effects of NUPR1 overexpression in both assays (Figure 5D,E). For comparisons among multiple groups, one‐way analysis of variance (ANOVA), followed by Tukey's post hoc test was applied.

### 3.5 Correlation Between NUPR1 Expression and Immune Infiltration

**FIGURE 6:**
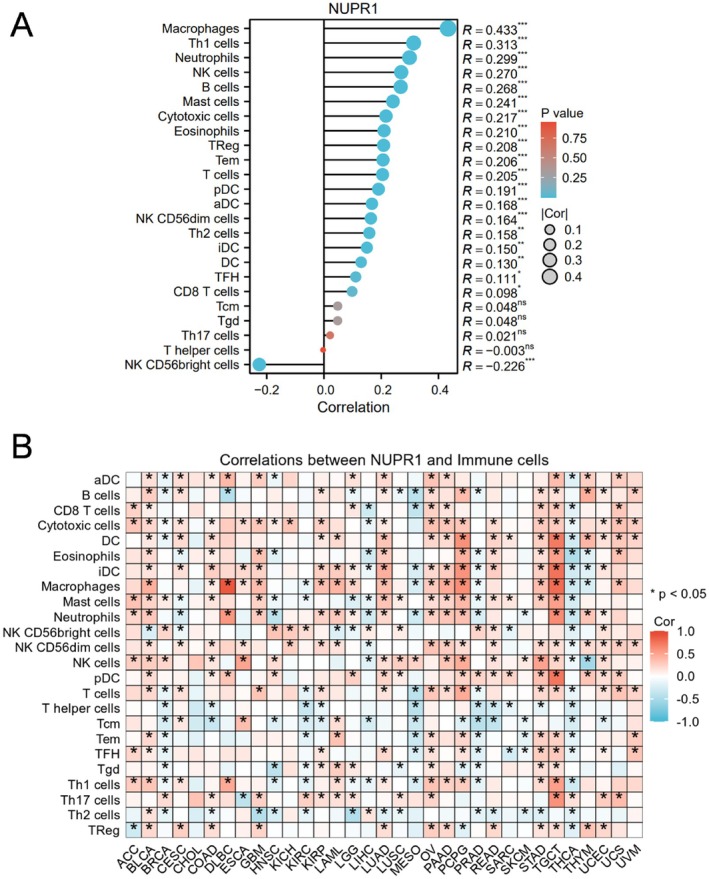
NUPR1 expression is associated with immune cell infiltration. (A) BLCA‐specific lollipop plot showing Spearman correlations between NUPR1 expression and 24 immune cell subsets; dot size denotes |correlation| and colour denotes *p* value. (B) Pan‐cancer heatmap depicting the correlations between NUPR1 expression and immune cell infiltration across cancer types; asterisks indicate *p* < 0.05.

To explore the immunological relevance of NUPR1, we analysed the correlation between NUPR1 expression and immune cell infiltration. BLCA‐specific lollipop plots revealed significant positive correlations between NUPR1 and M2‐like macrophages, regulatory T cells, and other immunosuppressive subsets. Consistently, a pan‐cancer heatmap showed broad associations of NUPR1 with immunosuppressive immune cells (Figure 6A,B).

### 3.6 NUPR1 Promotes M2 Macrophage Polarization In Vitro

**FIGURE 7:**
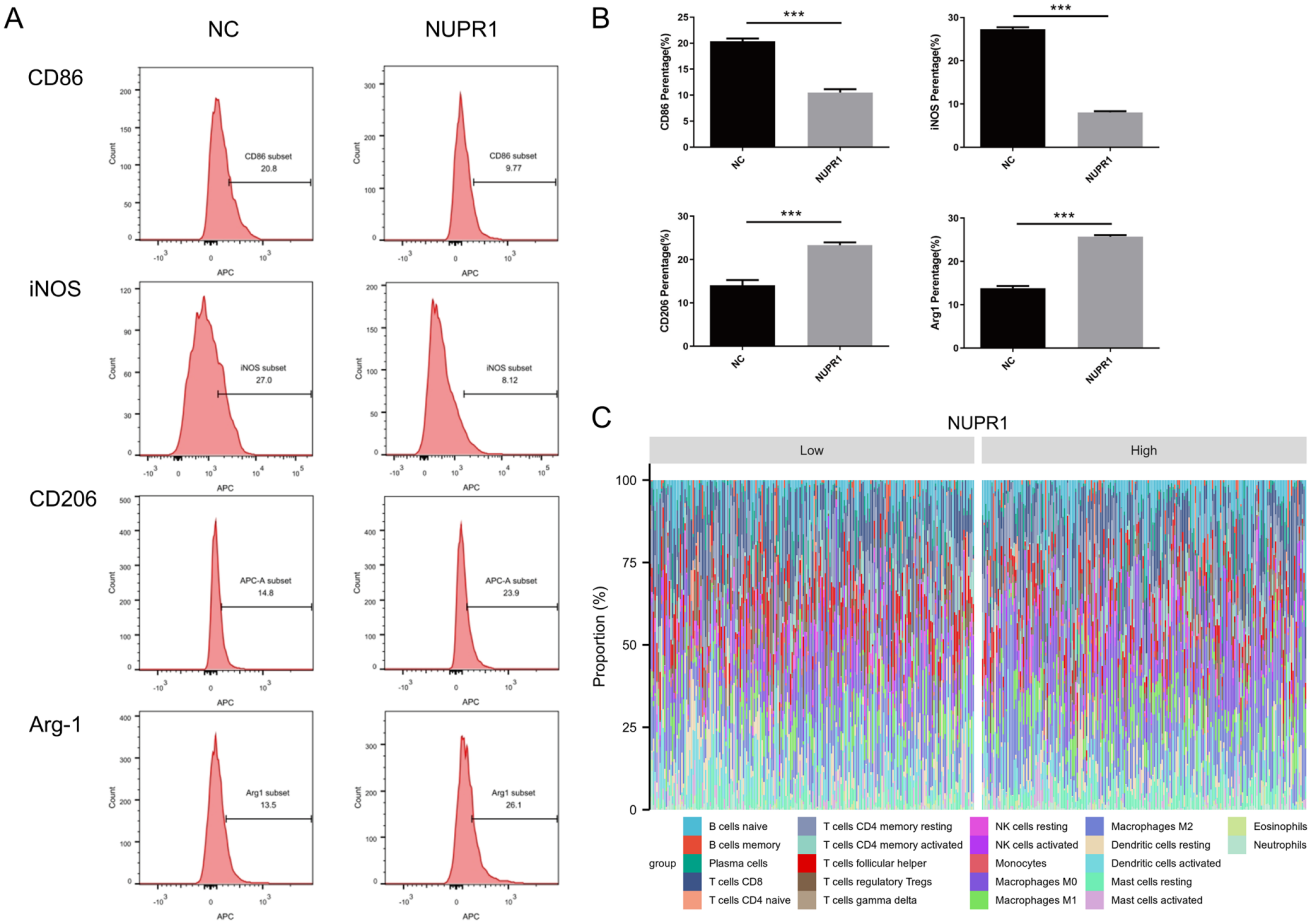
NUPR1 promotes M2 macrophage polarization. (A) Representative flow‐cytometry histograms of M1 markers (CD86, iNOS) and M2 markers (CD206, Arg‐1) in THP‐1‐derived macrophages exposed to supernatants from NUPR1‐overexpressing or control cells. (B) Quantification of positive fractions for each marker. Data are presented as mean ± SD (or SEM, as indicated). Statistical significance was determined using one‐way ANOVA, followed by Tukey's post hoc test. Significance levels: *p < 0.05, **p < 0.01, ***p < 0.001. (C) Stacked bar plot showing immune‐cell composition stratified by low versus high NUPR1 expression.

We next assessed whether NUPR1 directly influences macrophage polarization. Flow cytometry and WB assays revealed that NUPR1 overexpression enhanced CD206/Arg1 expression and reduced CD86/iNOS levels, indicating a shift towards M2 polarization (Figure 7A,B). Conversely, modulation of autophagy altered this effect. Visualization by stacked histogram further highlighted the increased M2 ratio under NUPR1 overexpression (Figure 7C).

### 3.7 Proposed Mechanistic Model

**FIGURE 8:**
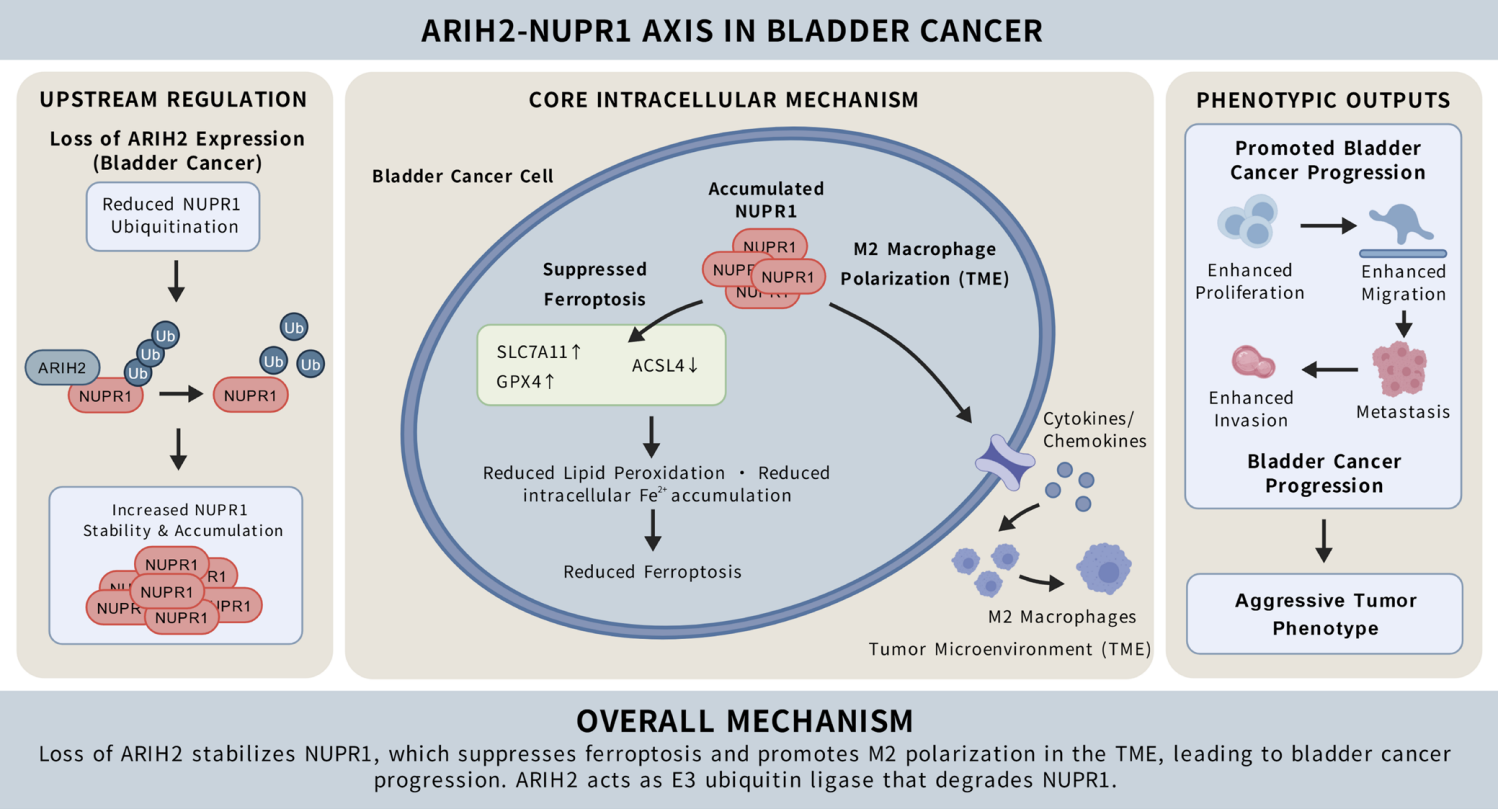
Schematic model of the ARIH2–NUPR1 axis in bladder cancer. Downregulation of ARIH2 decreases the ubiquitination and increases the stability of NUPR1. Elevated NUPR1 upregulates SLC7A11/GPX4 and downregulates ACSL4 to reduce lipid peroxidation and ferroptosis, and promotes M2 macrophage polarization, collectively facilitating tumour proliferation, migration and metastasis.

A schematic model was constructed to summarize these findings (Figure 8). Downregulation of ARIH2 reduces the ubiquitination of NUPR1, thereby stabilizing and increasing NUPR1 protein levels. Elevated NUPR1 suppresses ferroptosis by upregulating SLC7A11 and GPX4 and downregulating ACSL4, which decreases lipid peroxidation and iron accumulation, thereby limiting ferroptotic cell death. At the same time, high NUPR1 promotes M2 macrophage polarization within the tumour microenvironment, enhancing immunosuppressive infiltration. Together, ARIH2 loss leads to NUPR1 accumulation, ferroptosis resistance, increased M2 polarization, and ultimately the promotion of bladder cancer progression.

## 4 Discussion

Bladder cancer remains a clinically challenging malignancy due to frequent recurrence, progression, and limited durable responses to current systemic therapies. In this study, we identify ARIH2 as a key E3 ligase that destabilizes NUPR1 via ubiquitination, thereby promoting ferroptosis and restraining M2‐like macrophage polarization, providing a mechanistic link between ubiquitin signalling, ferroptosis resistance, and immune modulation.

NUPR1 was the focus of our study. Recent studies have shown that NUPR1 is closely related to the occurrence and progression of tumours. In colon cancer, the knockdown of NUPR1 resulted in decreased tumour cell growth, decreased colony formation and increased apoptosis [17]. In thyroid cancer, immunohistochemical staining of NUPR1 in tumour tissues was deeper than that in normal tissues [18]. In lung cancer, patients with high levels of NUPR1 in tumour tissues have significantly shorter overall survival [19]. In our previous study, we showed that high expression of NUPR1 can promote the proliferation, migration, invasion and apoptosis of bladder cancer cells both in vitro and in vivo [8, 20, 21]. To further explore the mechanism of NUPR1 in bladder cancer, we first identified the proteins that bind NUPR1 via IP–MS in T24 cells overexpressing NUPR1. Among them, three ubiquitination‐related proteins (RCN2, TXNRD2 and ARIH2) were identified. Ubiquitination is a common posttranslational modification process that has been shown to be involved in the occurrence and development of bladder cancer [22, 23]. Co‐IP and immunofluorescence experiments revealed that ARIH2 significantly interacts with NUPR1.

ARIH2 belongs to the E3 ubiquitin ligases and plays a role in transferring ubiquitin from E2 to substrate proteins via ubiquitination. E3 ubiquitin ligases are closely related to the tumorigenesis, progression and drug resistance of bladder cancer [24]. Targeting E3 ubiquitin ligases has been shown to be a novel approach for the treatment of bladder cancer, but whether ARIH2 plays a role in bladder cancer is currently not known [25]. Notably, ARIH2 has also been implicated in p53‐associated stress responses. Previous work reported that ARIH2 can facilitate Nutlin‐3a–triggered p53 activation and enhance tumour cell death under p53‐activating conditions. This raises the possibility that ARIH2 may exert tumour‐suppressive effects through coordinated regulation of multiple stress‐response pathways, including p53 signalling and NUPR1‐dependent ferroptosis resistance. Although p53 activity was not directly assessed in the current study, integrating p53‐related signalling into the ARIH2–NUPR1 framework may provide additional mechanistic insight and will be explored in future studies. In this study, ARIH2 was knocked down or overexpressed in bladder cancer cell lines to investigate the functional changes in bladder cancer cells. The results showed that ARIH2 knockdown enhanced the proliferation and migration ability of bladder cancer cells, decreased the expression of E‐cadherin, and increased the expression of N‐cadherin and vimentin. The expression of the autophagy‐related proteins PINK1, Parkin and LC‐3I/II was increased, and the number of apoptotic cells was significantly decreased. After ARIH2 overexpression, the proliferation and migration ability of bladder cancer cells decreased, and the number of apoptotic cells significantly increased, which indicated that ARIH2 indeed inhibited the progression of bladder cancer. We subsequently verified the regulatory effect of ARIH2 on NUPR1 ubiquitination by adding CHX to ARIH2‐knockdown T24 cells and detecting the level of NUPR1 ubiquitination via IP. The results revealed that the level of ubiquitinated NUPR1 was significantly reduced and that the protein content of NUPR1 was increased. These results indicated that ARIH2 knockdown could maintain the stability of NUPR1 by reducing the ubiquitination of NUPR1 in bladder cancer cells, thereby affecting the function of these cells.

In our previous study, bioinformatics analysis revealed that NUPR1 expression was associated with ferroptosis and macrophage polarization in bladder cancer. Second, Western blotting was used to detect the expression of ferroptosis‐related proteins in bladder cancer cells, and the results revealed that the expression of ACSL4 was significantly increased and that the expression of SLC7A11 and GPX4 was significantly decreased in the NUPR1‐knockdown group. The significant reduction in ACSL4 protein expression and significant increase in SLC7A11 and GPX4 protein expression in the NUPR1 overexpression group are consistent with previous findings that NUPR1‐mediated LCN2 expression prevents ferroptosis‐related cell death by reducing iron accumulation and subsequent oxidative damage [26]. Importantly, these conclusions were further supported by functional ferroptosis readouts, as C11‐BODIPY staining and Fe^2+^ quantification consistently reflected ferroptosis suppression upon NUPR1 upregulation and the opposite changes after NUPR1 depletion. Additionally, emerging evidence highlights the role of non‐coding RNAs in modulating NUPR1 activity. For instance, the ferroptosis‐related lncRNA AL136084.3 has been shown to directly bind NUPR1, potentially influencing its stability or transcriptional activity in bladder cancer [27]. This interaction suggests a multi‐layered regulatory mechanism of NUPR1 in ferroptosis suppression. Tumour‐associated macrophages (TAMs) are the main components of the tumour microenvironment and key cells that regulate tumour development, metastasis, the immune response, inflammation and chemoresistance [28, 29]. In response to the tumour microenvironment (TME) stimulation, circulating monocytes are recruited and differentiated into TAMs, most of which are defined as the alternative activation (M2) phenotype and produce an immunosuppressive TME to support tumour progression [30]. Current studies have suggested that autophagy contributes to TME regulation by TAMs. Although the specific mechanism is still unclear, it has been confirmed that autophagy can downregulate the expression of major histocompatibility complex (MHC) on macrophages, thereby reducing their ability to present tumour antigens [31]. In addition, the activation of autophagy in TAMs may promote tumour‐related inflammation, and the proinflammatory cytokine IL1β promotes its transport and release from macrophages through secretory autophagy [32]. Autophagy‐promoted secretion of inflammatory cytokines may increase the TME inflammatory response, thereby facilitating tumour progression. Our study demonstrated that NUPR1 promotes M2 polarization of macrophages, consistent with both immune correlation analysis and in vitro functional assays. These results provide ideas for therapeutic strategies involving autophagy in bladder cancer, but further studies are still needed.

Despite the mechanistic insights provided by this study, several limitations should be acknowledged. The current work was primarily conducted using a single bladder cancer cell line, and further validation in additional experimental models and in vivo systems will be required. Moreover, pharmacological modulation of ferroptosis and more sophisticated approaches to dissect tumour–macrophage interactions may further strengthen the causal links identified in this study.

In conclusion, this study demonstrated that ARIH2‐mediated ubiquitination destabilizes NUPR1, thereby promoting ferroptosis and restraining M2 macrophage polarization. These findings reveal potential therapeutic targets for bladder cancer by manipulating the ARIH2–NUPR1 pathway, offering new avenues for treatment strategies aimed at improving patient outcomes.

## Author Contributions

**Hannuo Deng:** methodology, investigation, writing – original draft, conceptualization, validation, visualization. **Yuchen Tan:** software, data curation, validation. **Lei Zhang:** supervision, funding acquisition, data curation. **Yuanyuan Mi:** conceptualization, methodology, investigation. **Zebin Shi:** writing – original draft, validation. **Li Zuo:** supervision, project administration. **Guangming He:** data curation, formal analysis, writing – review and editing. **Lifeng Zhang:** supervision, funding acquisition, writing – review and editing, project administration, resources. **Shenglin Gao:** supervision, resources. **Jie Peng:** supervision, resources.

## Funding

This work was supported by the Basic Research Project of Changzhou Medical Center (Grants CMCB202319 and CMCB202313), Science and Technology Project of Changzhou Health Commission (Grant ZD202440), Jiangsu Province 333 Project (Grant RC202202), Changzhou Health Committee Youth Science and Technology Program (Grant QN202227), Top Talent of Changzhou ‘The 14th Five‐Year Plan’ High‐Level Health Talents Training Project (Grants 2022CZBJ057 and 2022CZBJ058), Jiangsu Province Postdoctoral Research Foundation (Grant 2021K588C), Changzhou Sci&Tech Program (CJ20220146), and Postdoctoral Science Startup Foundation (Grants BSH202009 and BSH202214).

## Ethics Statement

The authors have nothing to report.

## Consent

The authors have nothing to report.

## Conflicts of Interest

The authors declare no conflicts of interest.

## Supporting information


**Figure S1:** Uncropped full‐length western blot images corresponding to the cropped blots shown in Figures 1, 2, 3, 4, 5.

## Data Availability

The data that support the findings of this study are available from the corresponding author upon reasonable request.
