# Supplementary material for: ARIH2 Ubiquitination Regulates NUPR1 to Inhibit Ferroptosis in Bladder Cancer
Source: J Cell Mol Med. 2026 Apr 17;30(8):e71147. doi: 10.1111/jcmm.71147 (PMC13090160; doi:10.1111/jcmm.71147)
Supplement: Supplementary file 1 — Figure S1: Uncropped full‐length western blot images corresponding to the cropped blots shown in Figures 1, 2, 3, 4, 5. [file JCMM-30-e71147-s001.docx]

Supplementary Figure S1.

Uncropped full-length western blot images corresponding to the cropped blots shown in Figures 1–5.


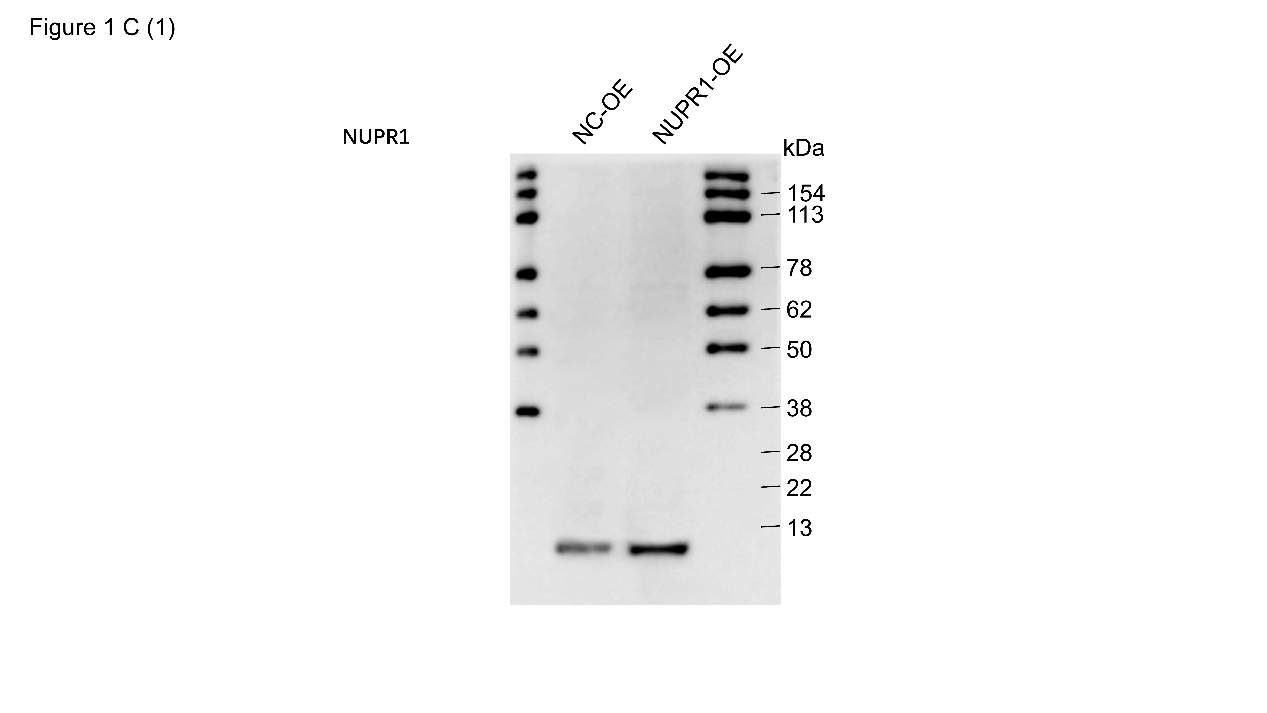

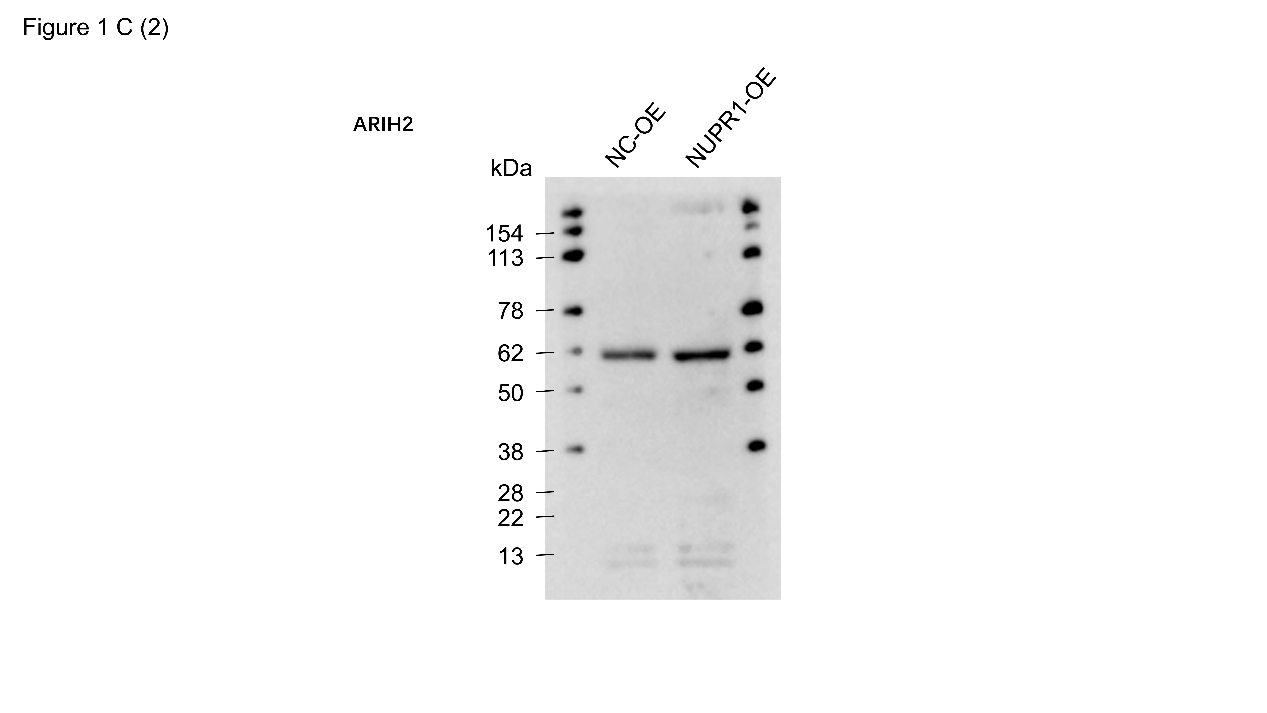
Figure1C IB NUPR1 Figure1C IB ARIH2

ARIH2

NUPR1


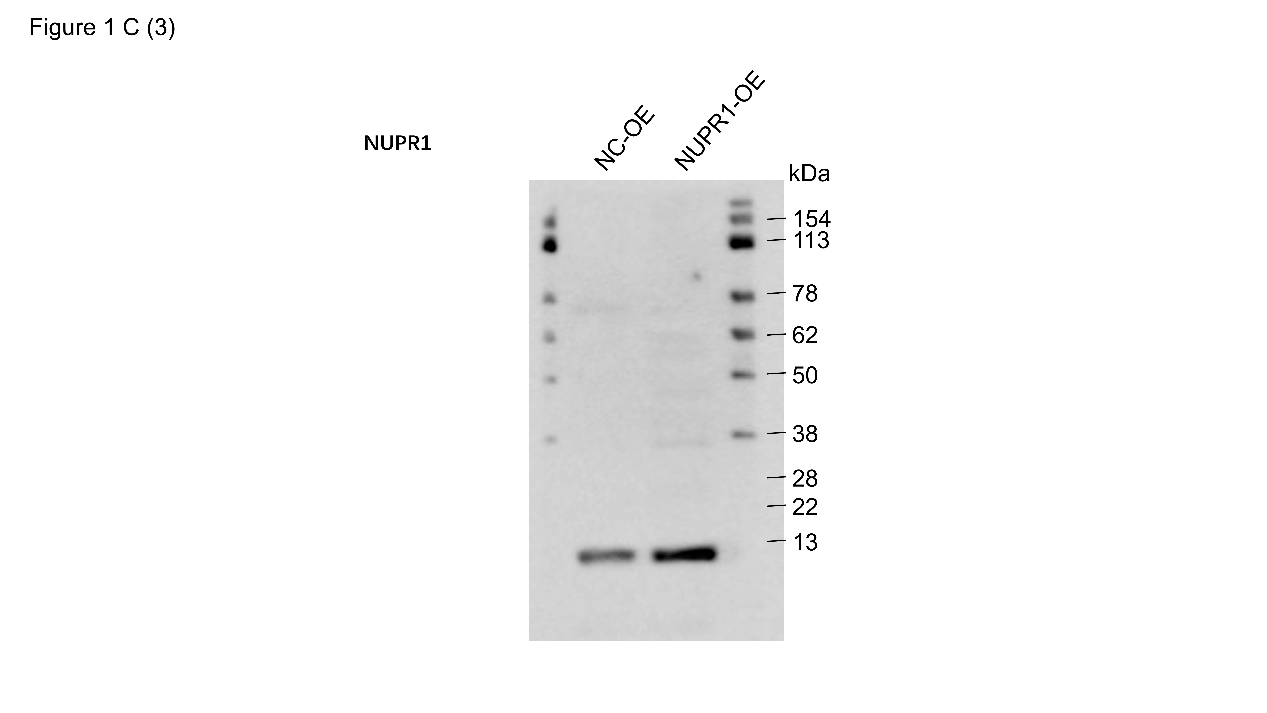

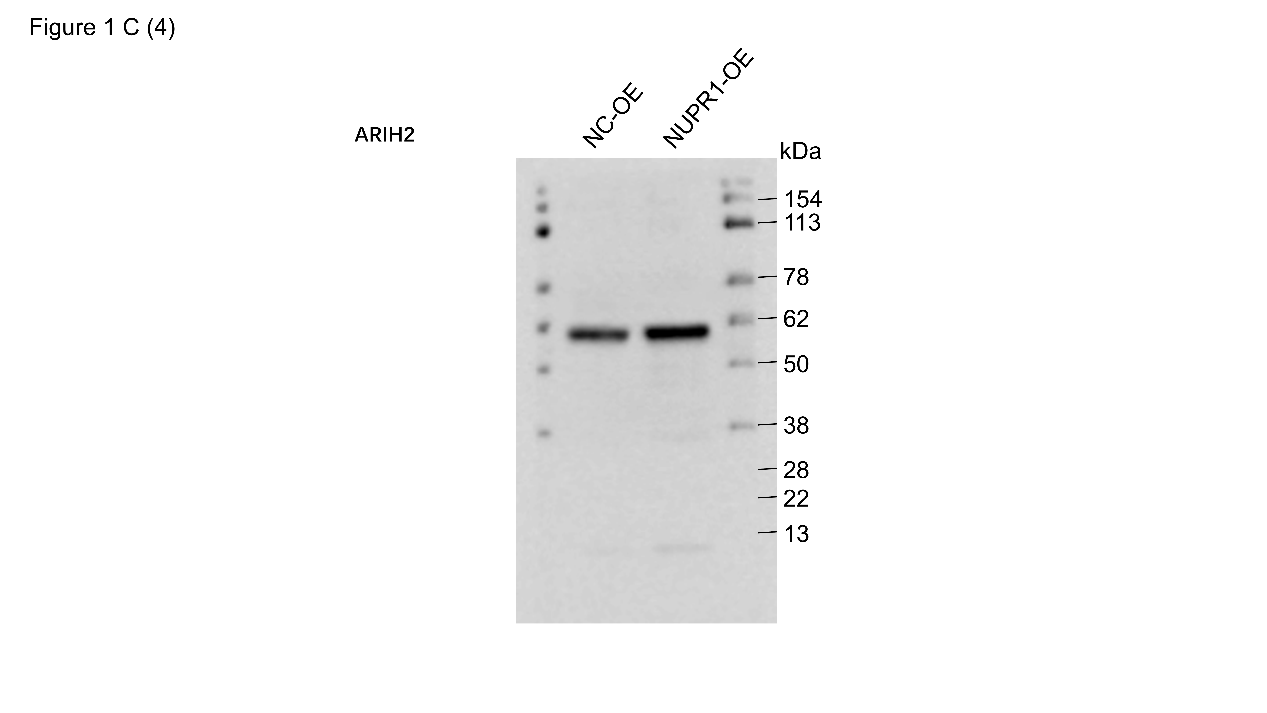
Figure1C Input NUPR1 Figure1C Input ARIH2

ARIH2

NUPR1


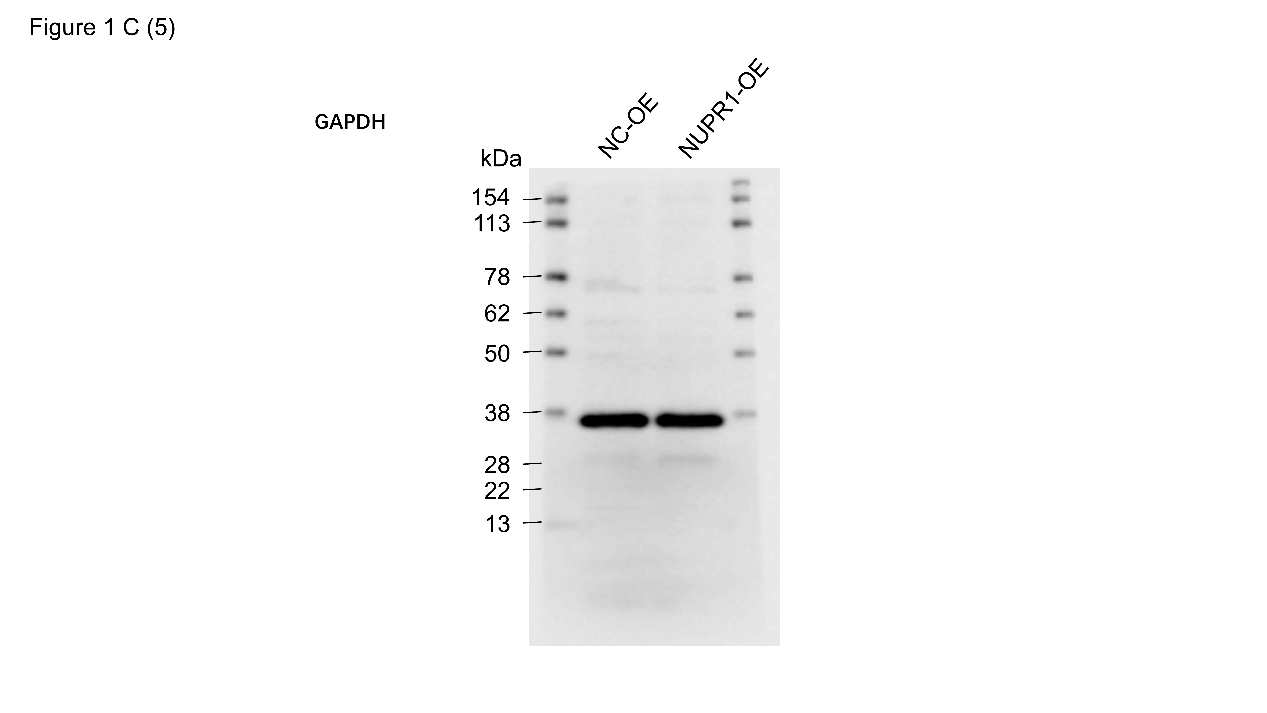
Figure1C Input GAPDH

GAPDH


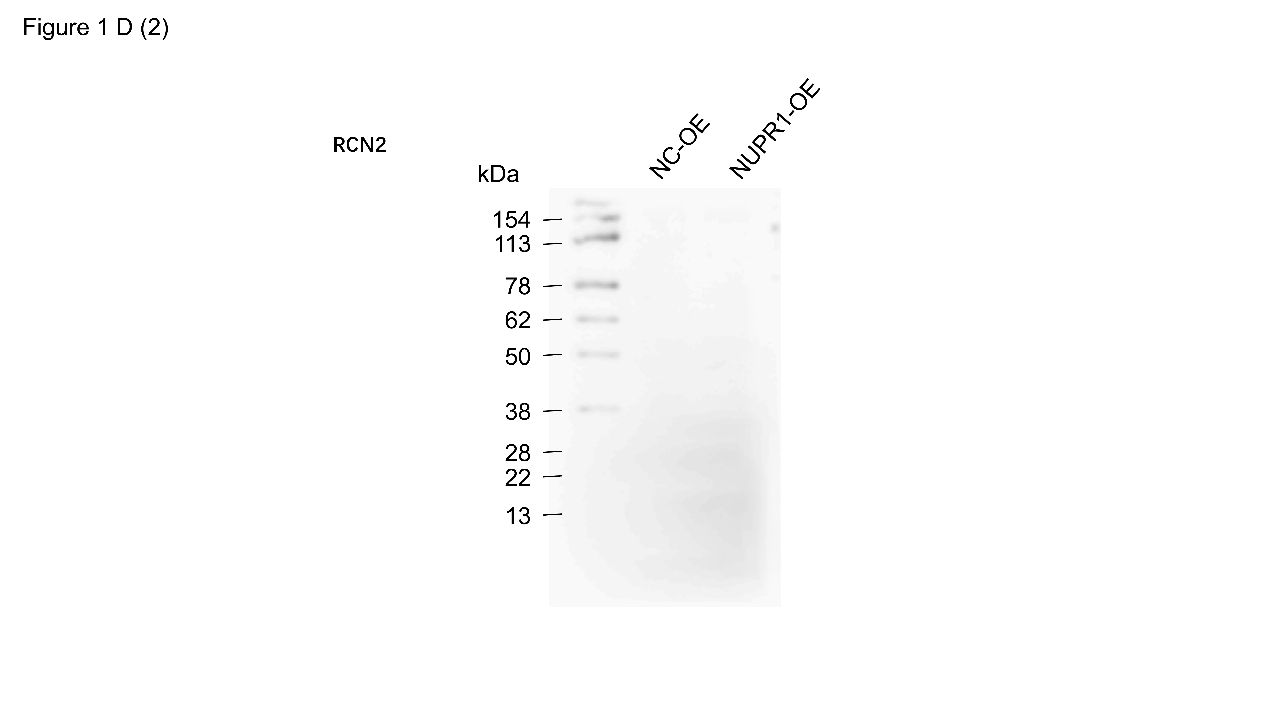

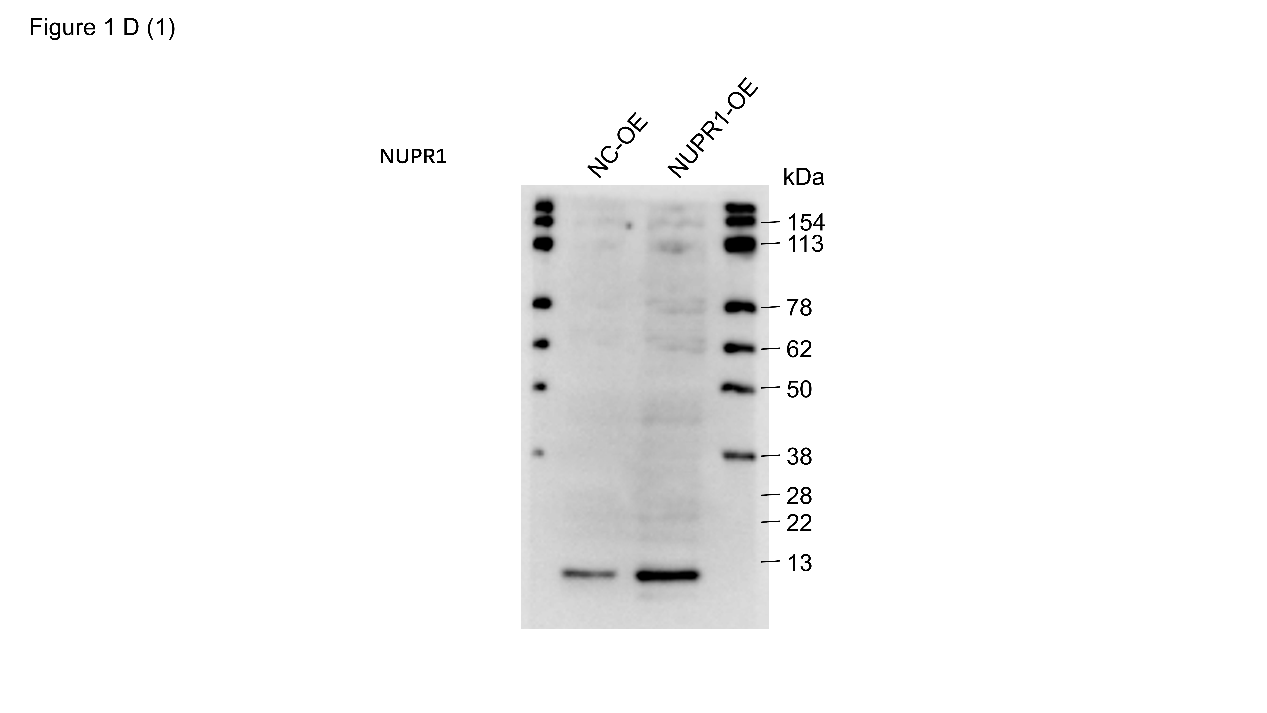
Figure1D IB NUPR1 Figure1D IB RCN2

RCN2 (kDa:55)

NUPR1


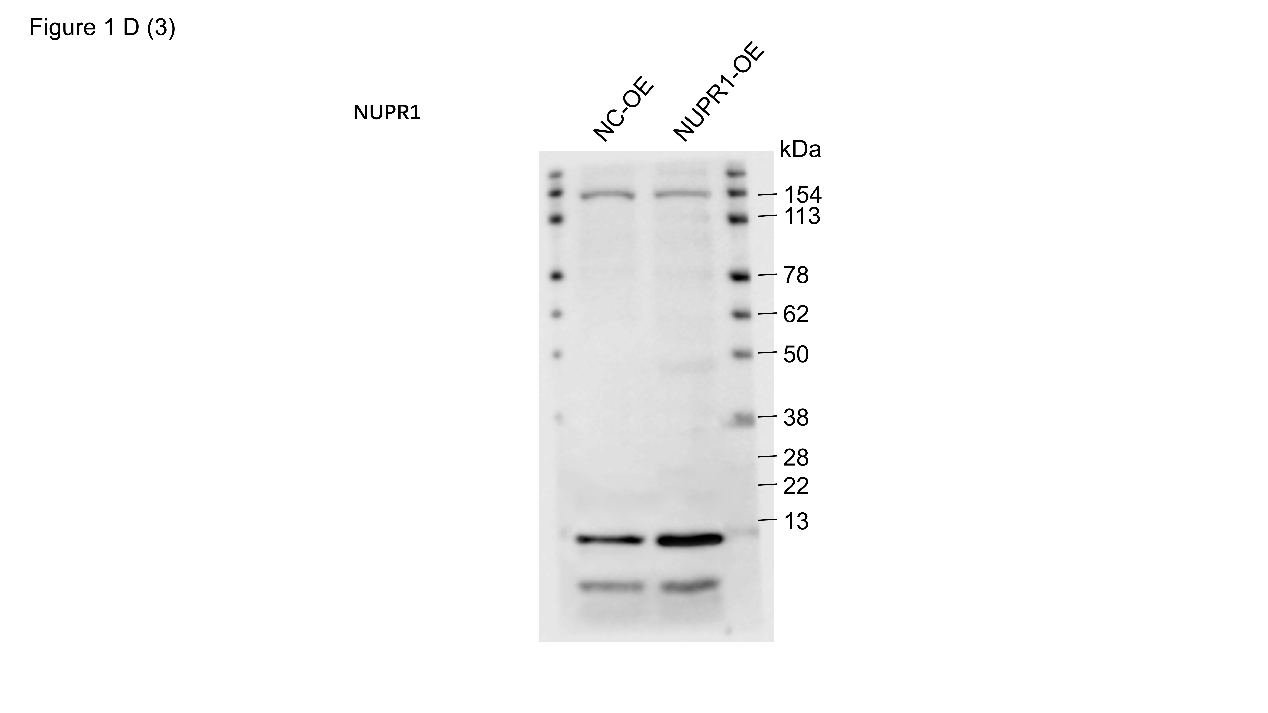

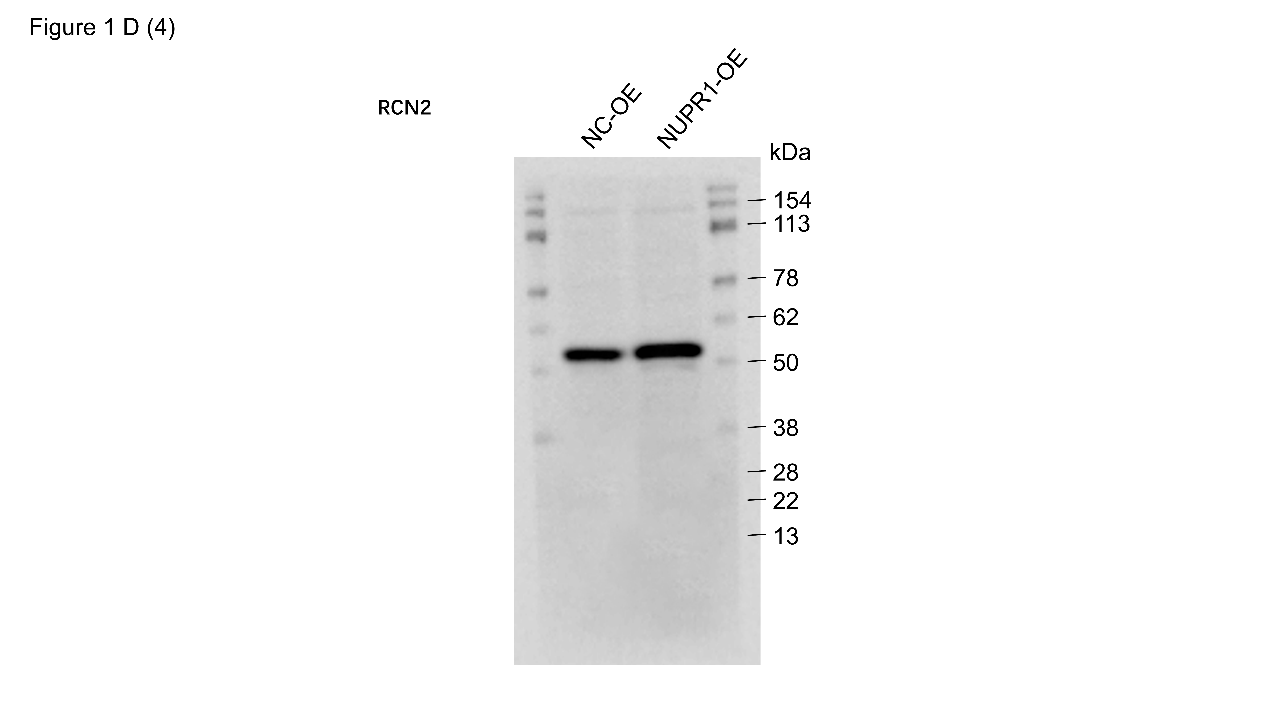
Figure1D Input NUPR1 Figure1D Input RCN2

RCN2

NUPR1


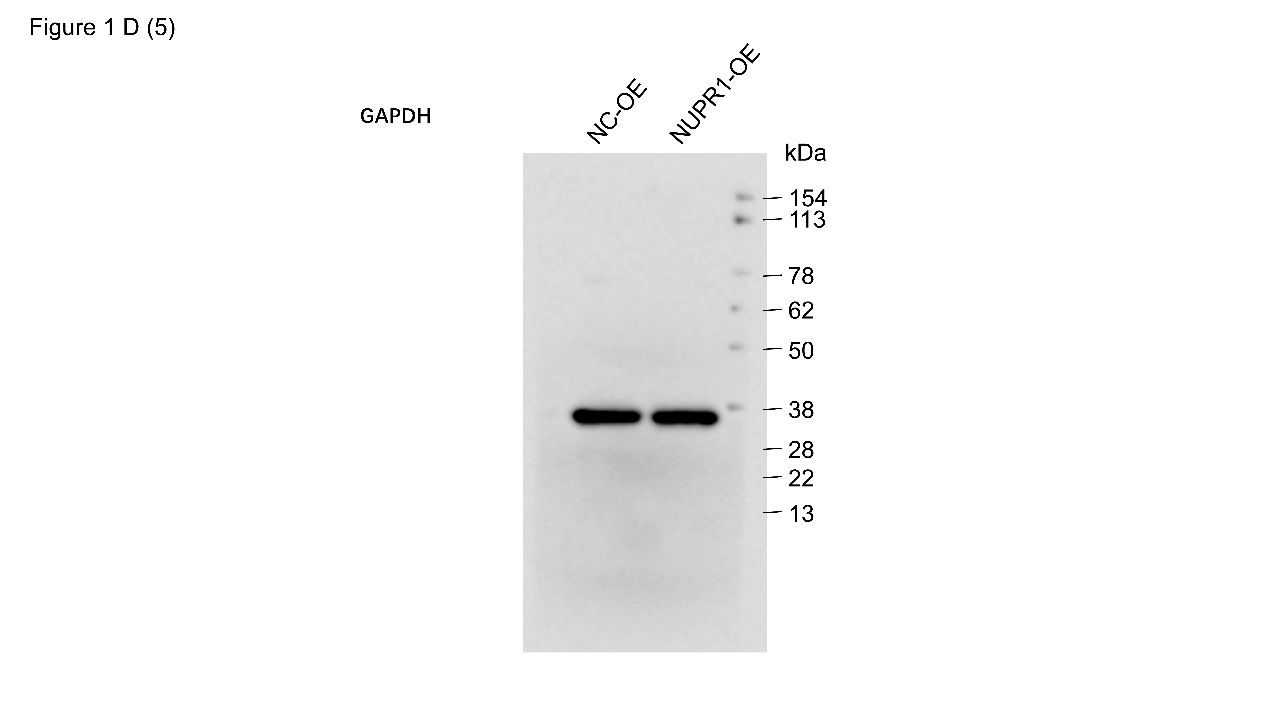
Figure1D Input GAPDH

GAPDH


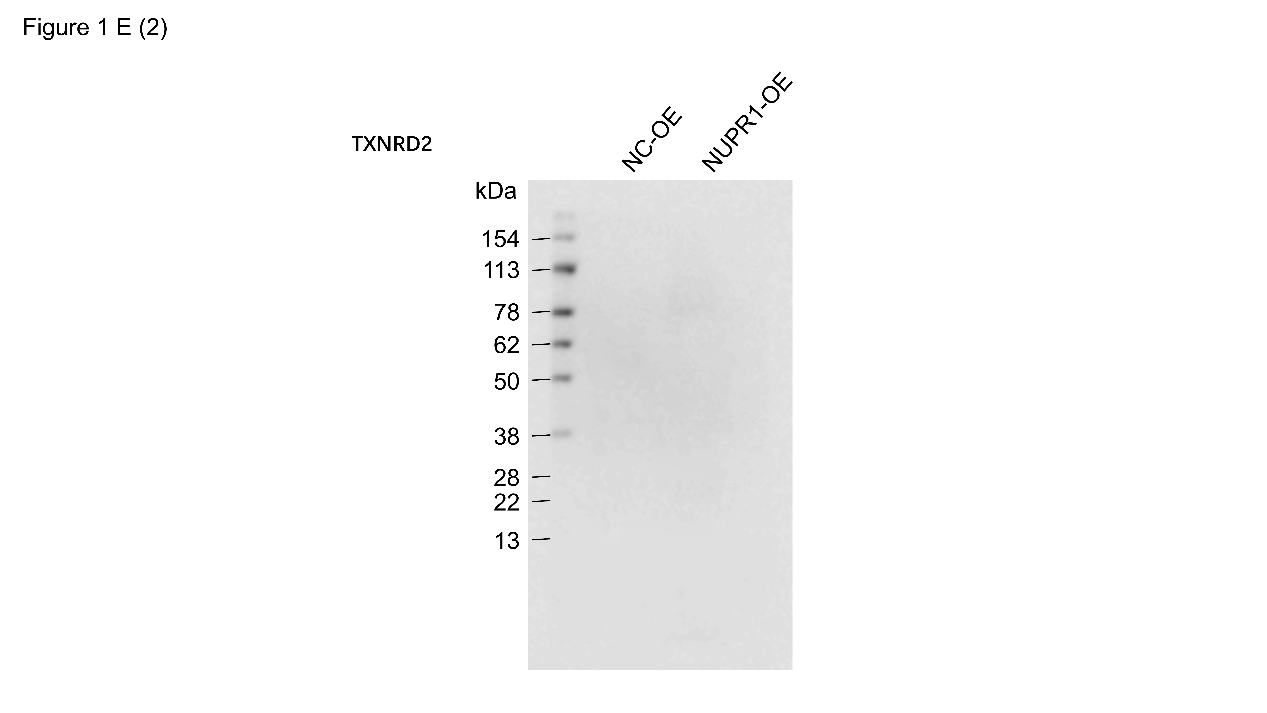

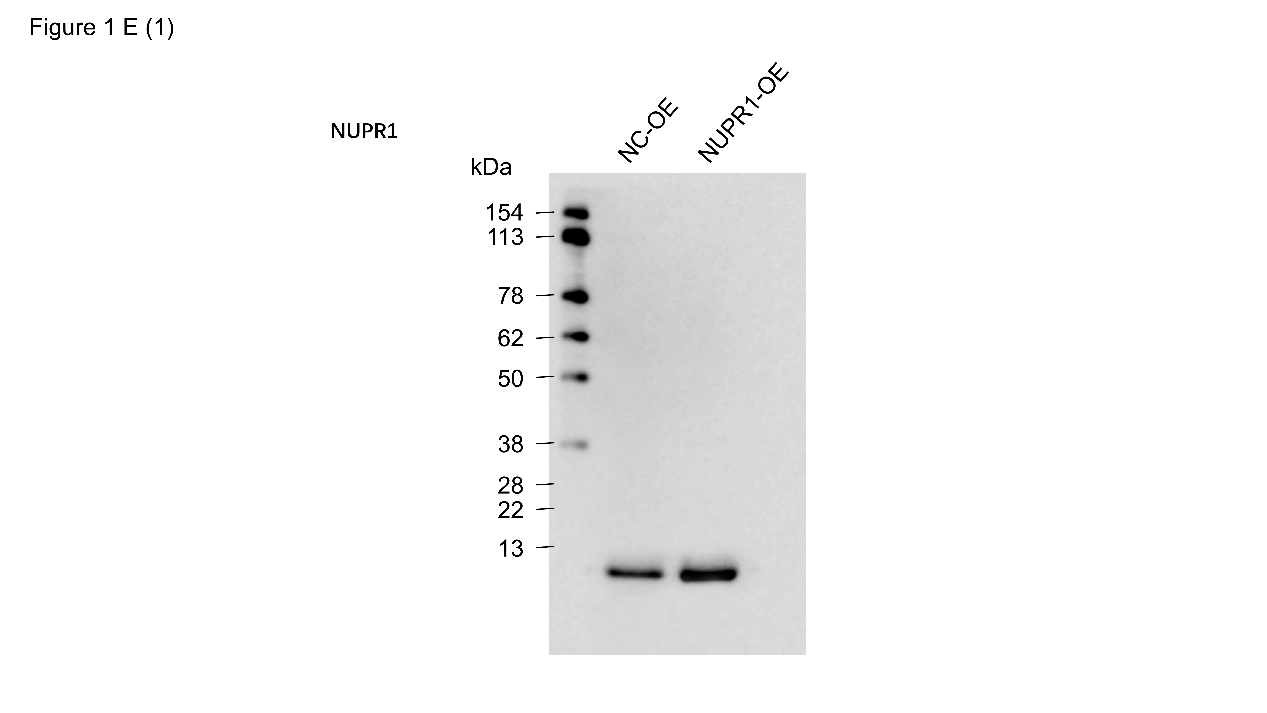
Figure1E IB NUPR1 Figure1E IB TXNRD2

TXNRD2 (kDa:54)

NUPR1


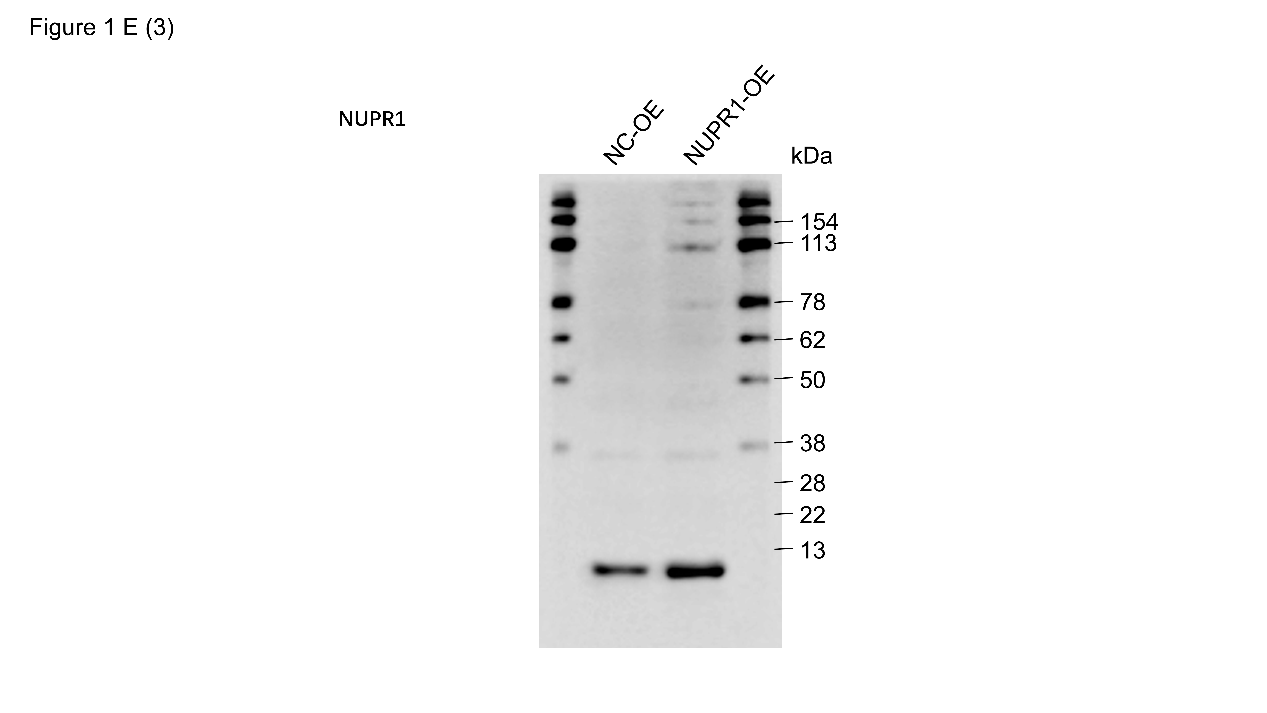

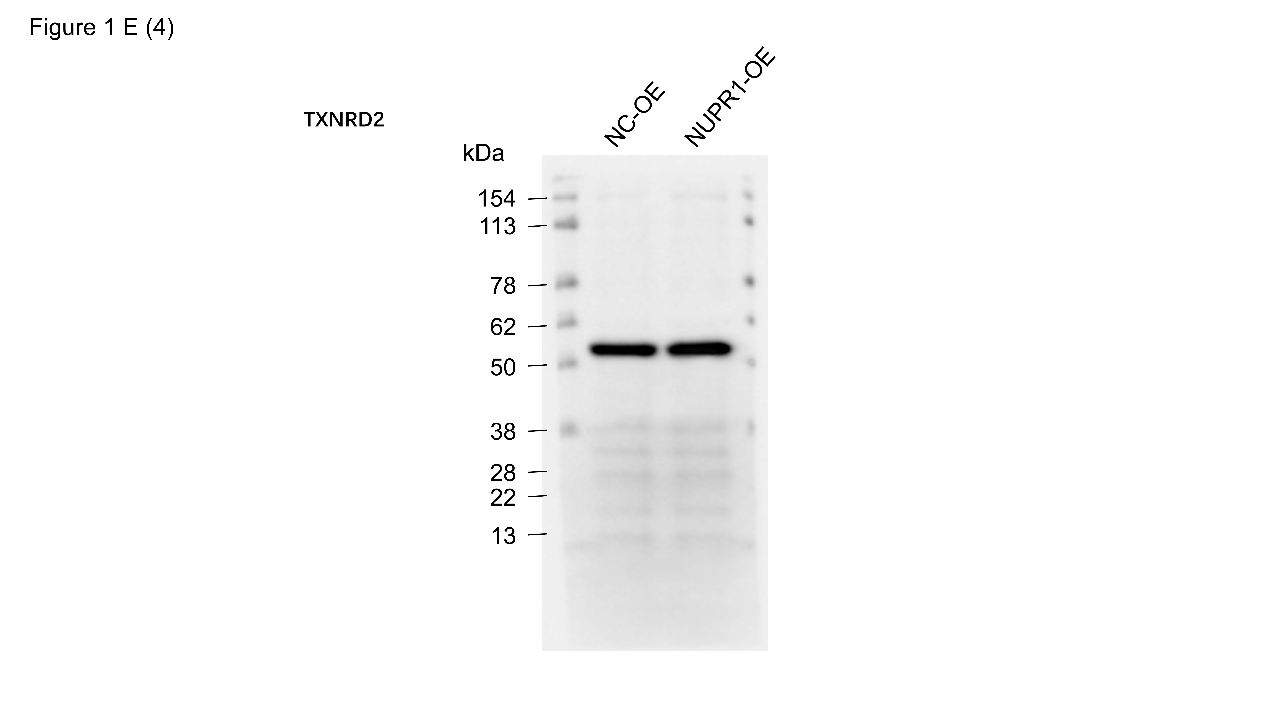
Figure1E Input NUPR1 Figure1E Input TXNRD2

TXNRD2

NUPR1


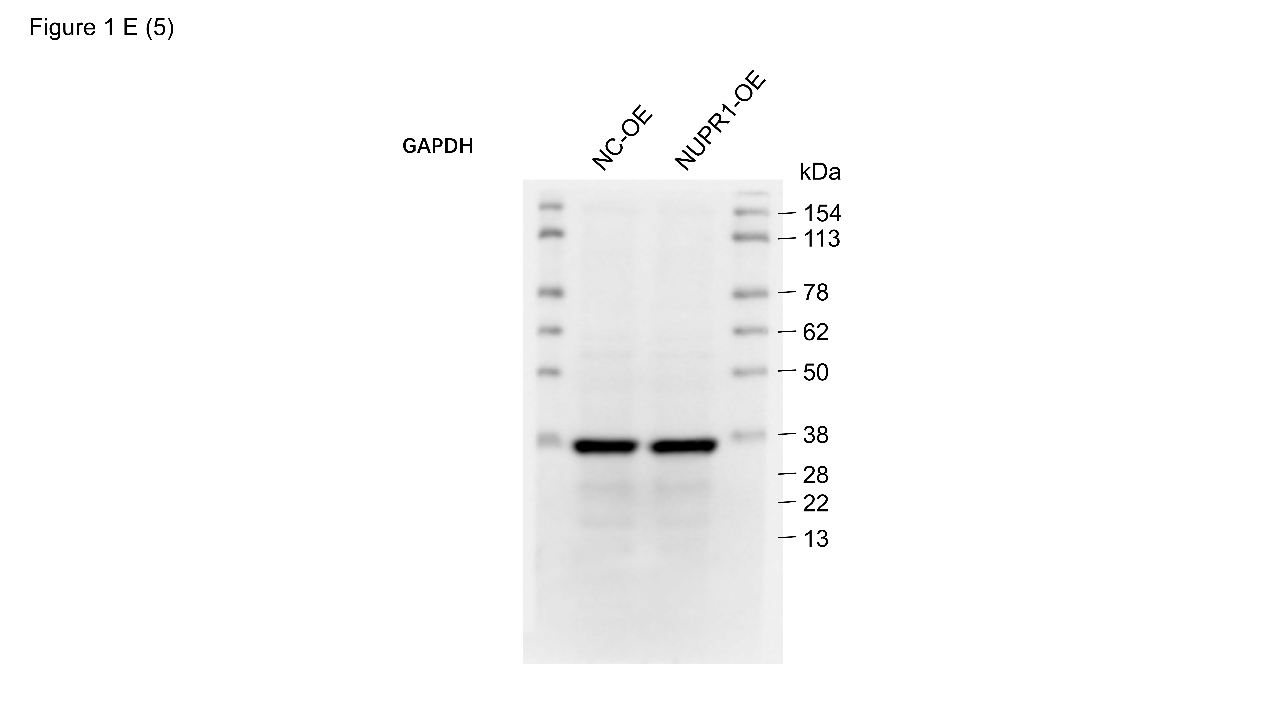
 Figure1E Input GAPDH

GAPDH


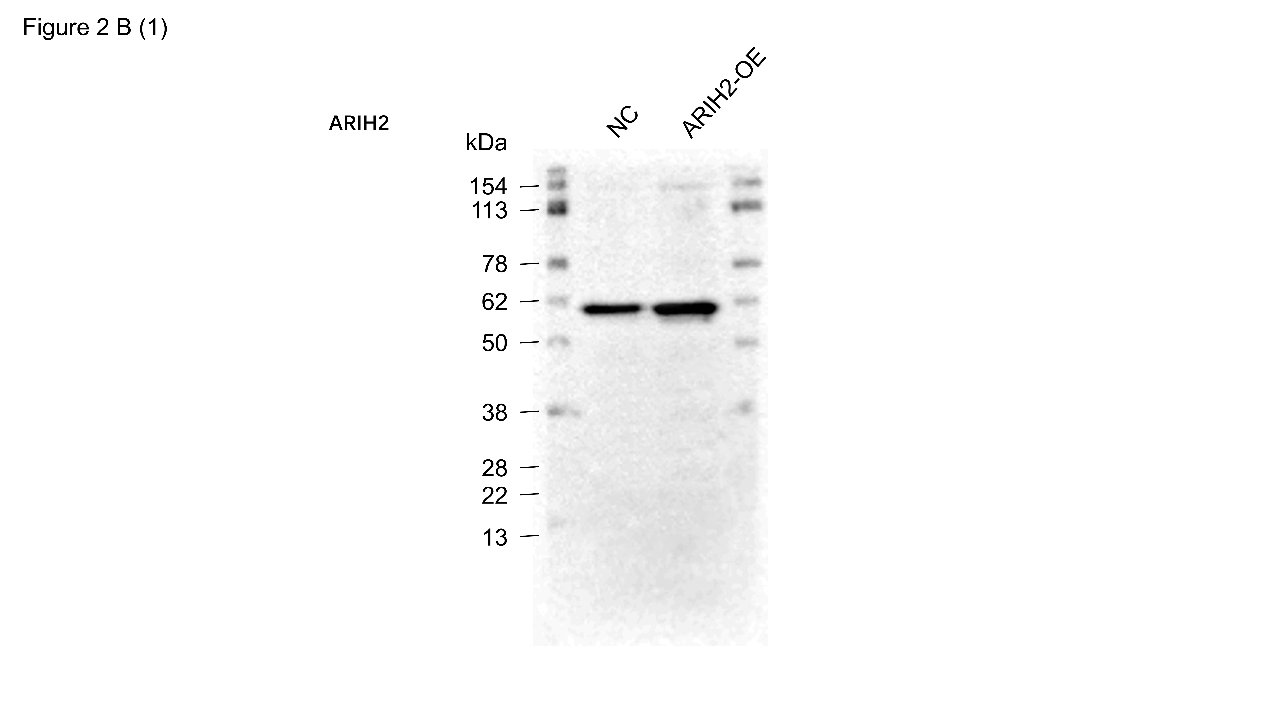

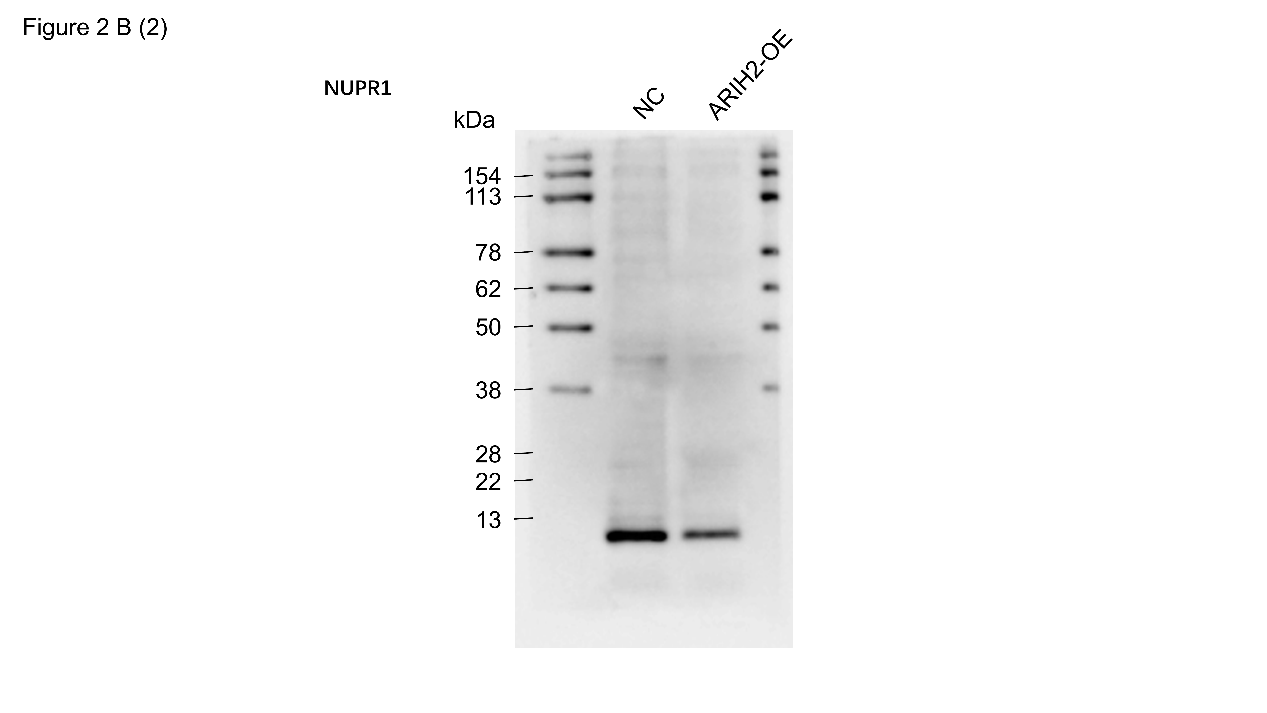
Figure2B ARIH2 Figure2B NUPR1

ARIH2

NUPR1


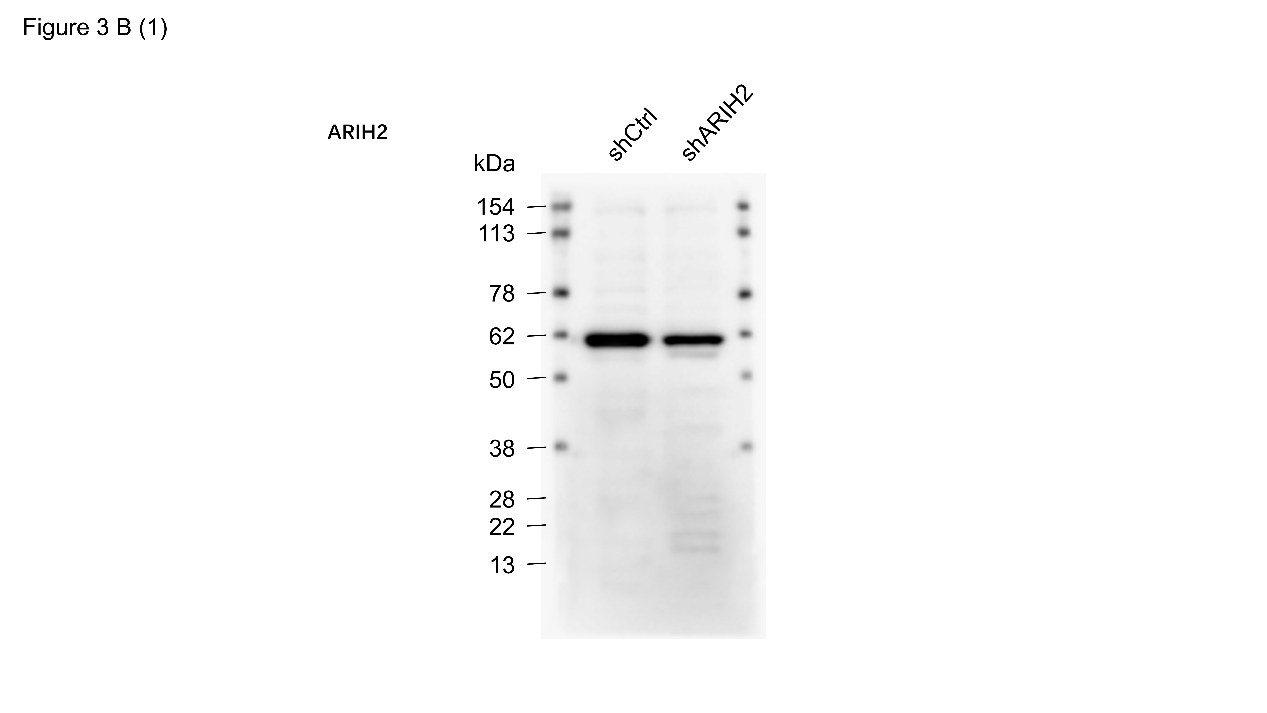

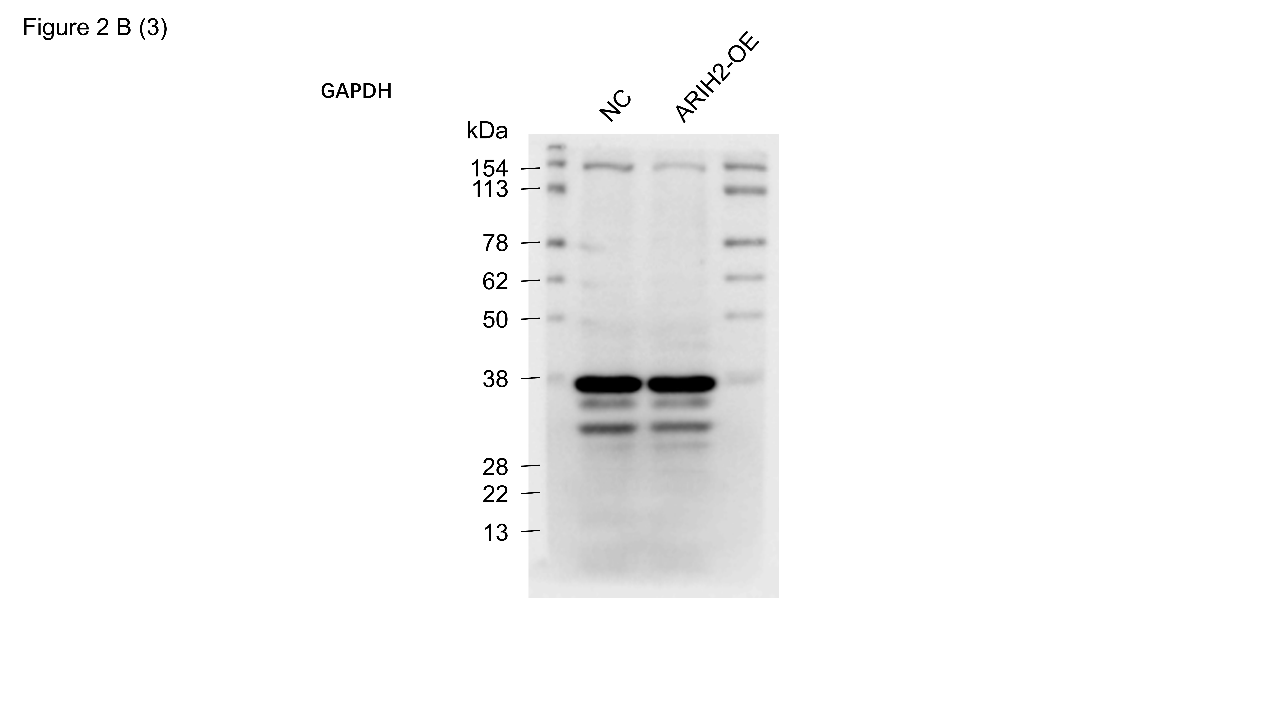
Figure2B GAPDH Figure3B ARIH2


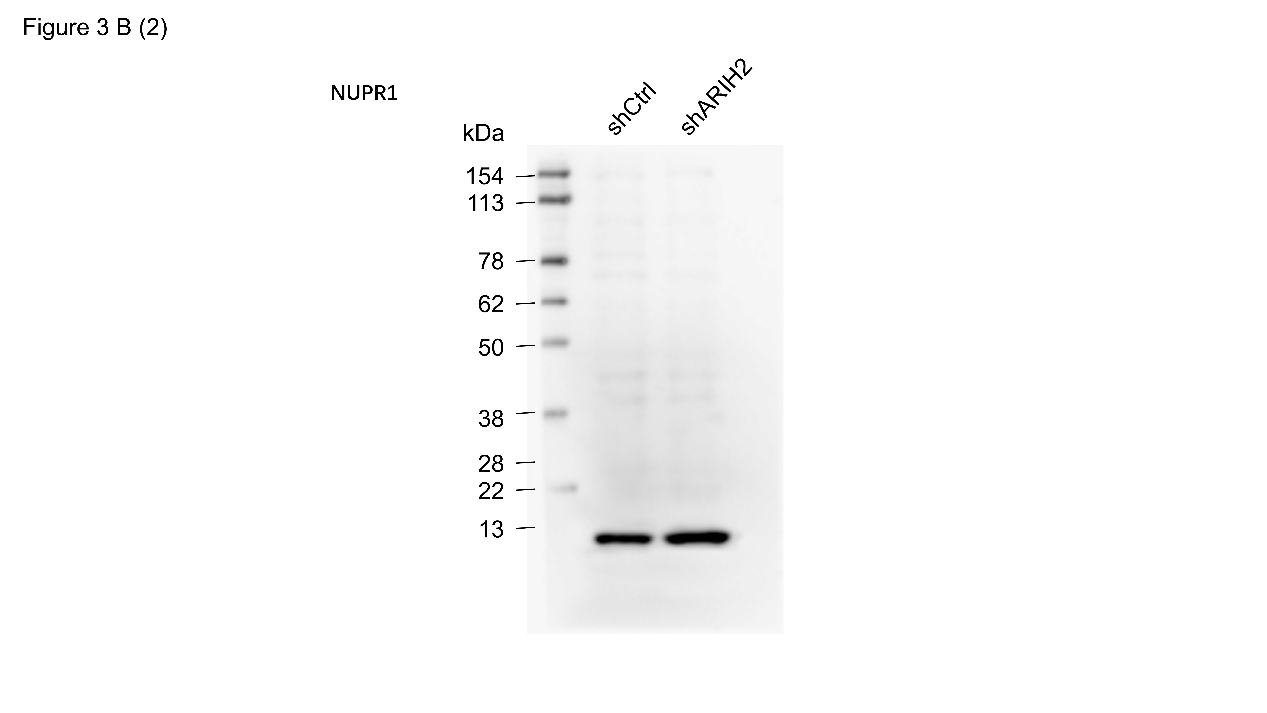


ARIH2

GAPDH

Figure3B NUPR1 Figure3B GAPDH


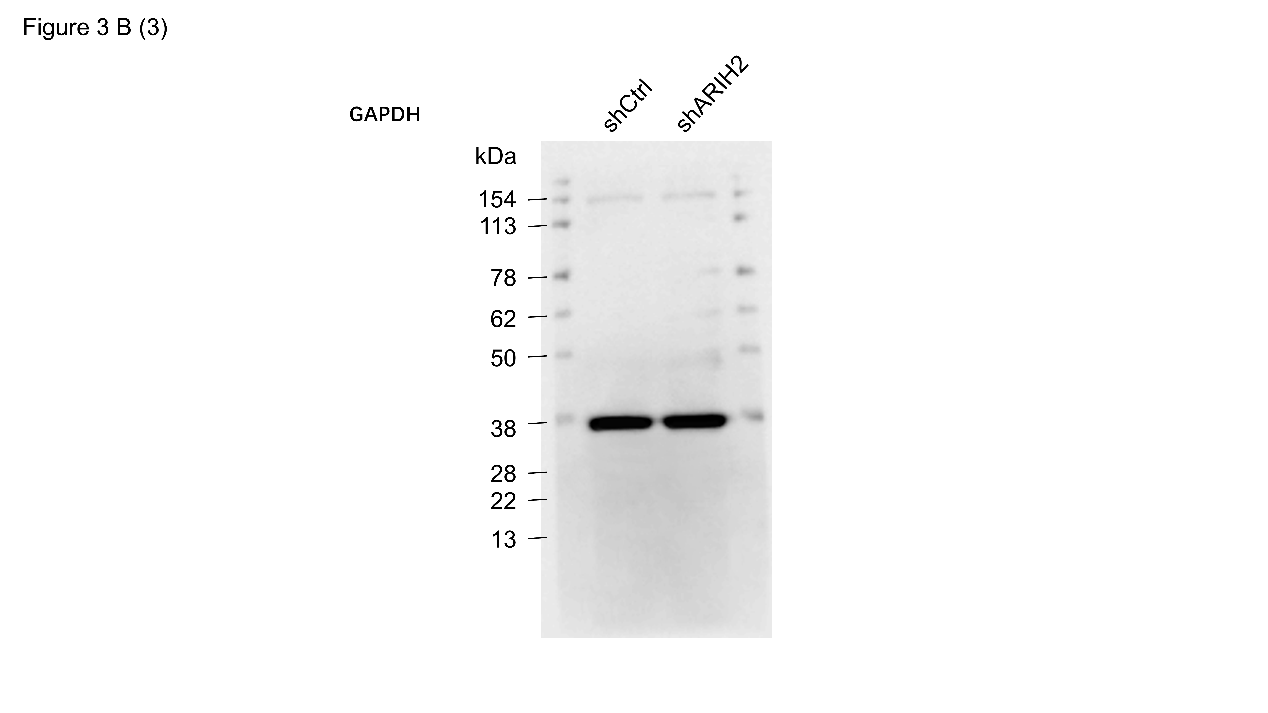


GAPDH

NUPR1


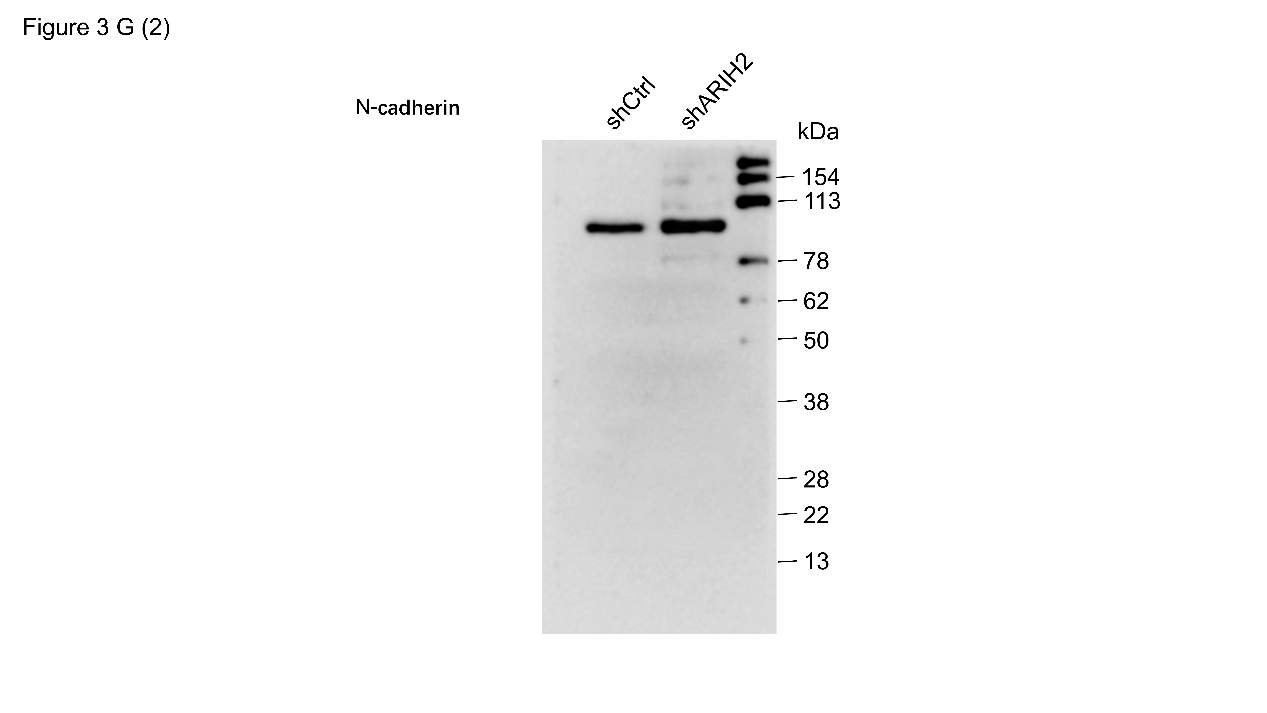

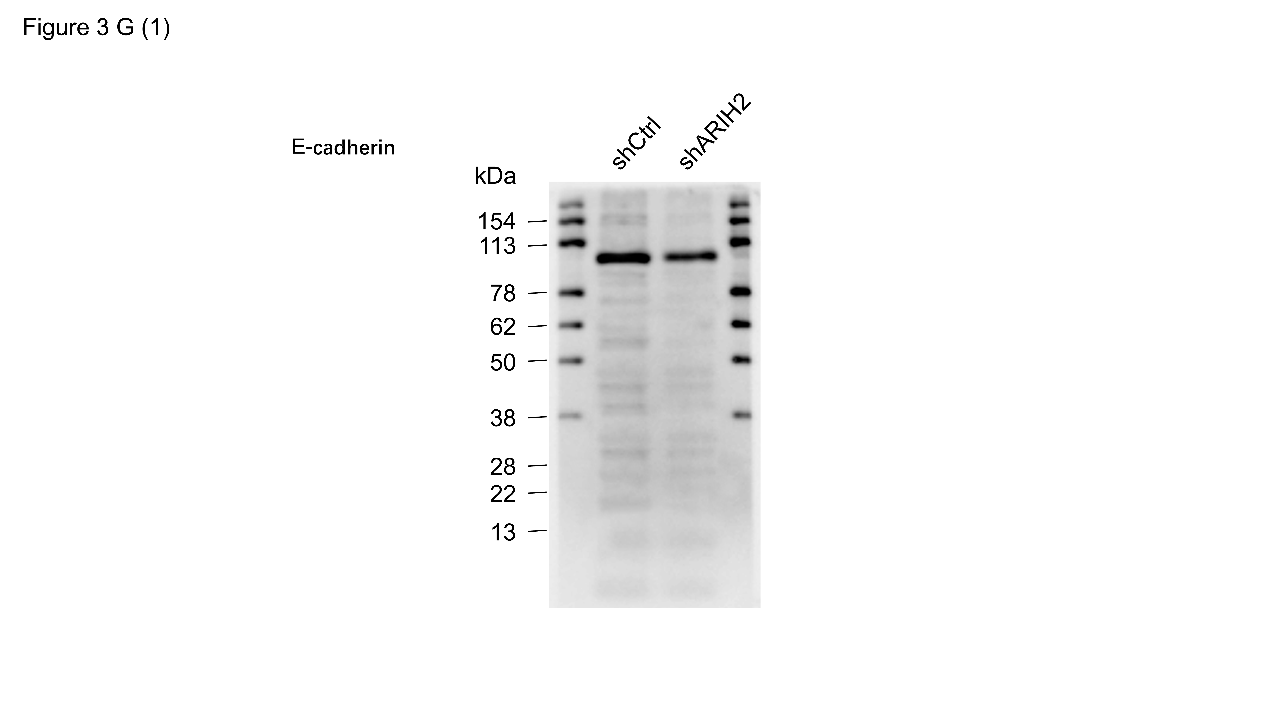
Figure4B E-cadherin Figure4B N-cadherin

N-cadherin

E-cadherin


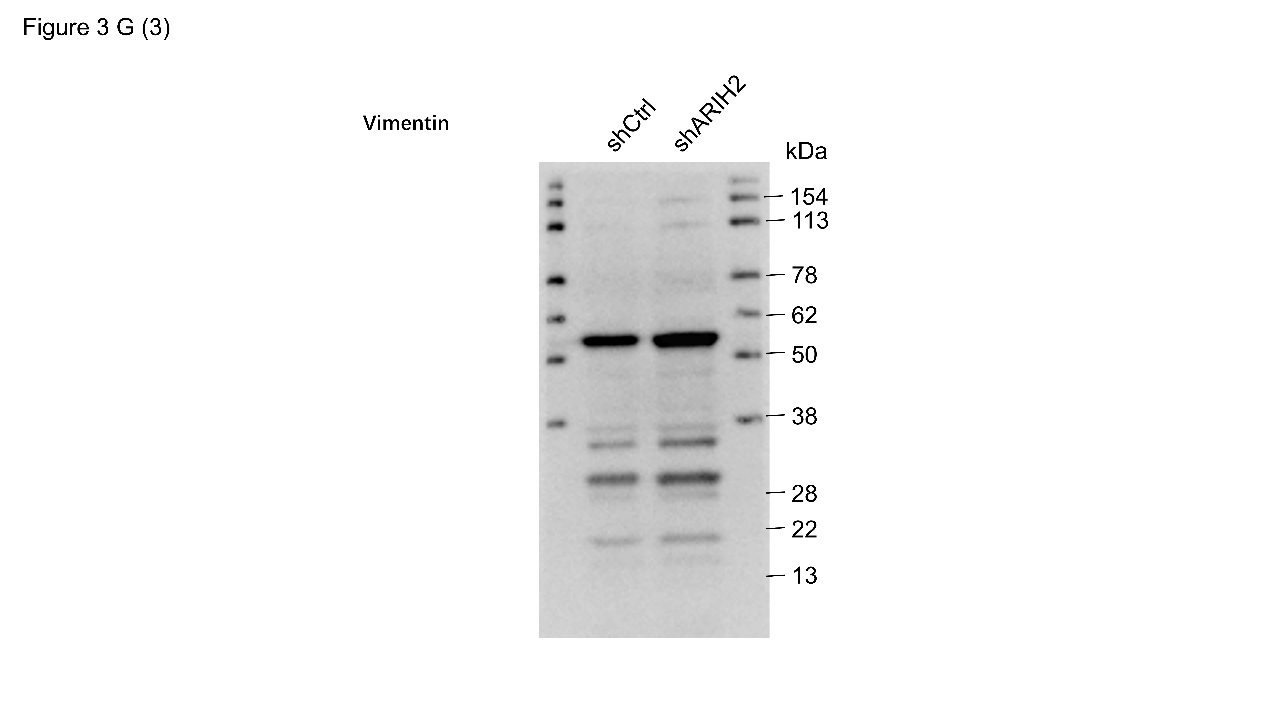


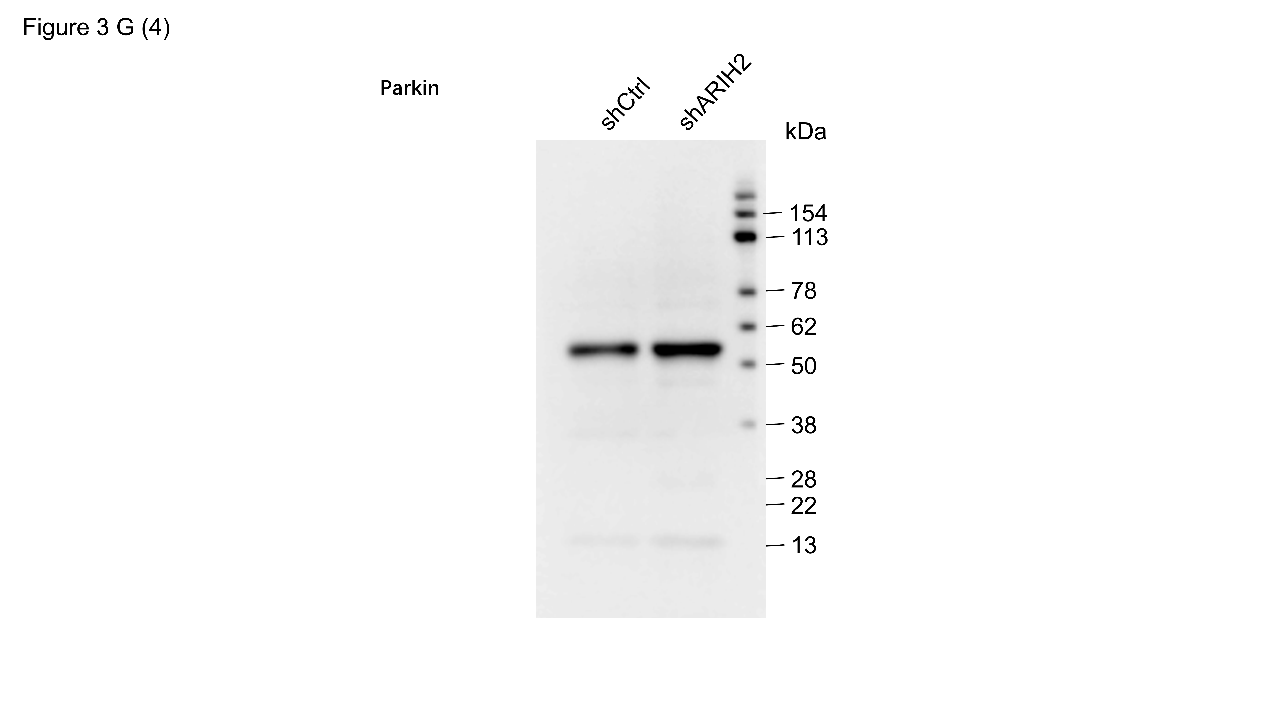
Figure4B Vimentin Figure4B Parkin

Parkin

Vimentin


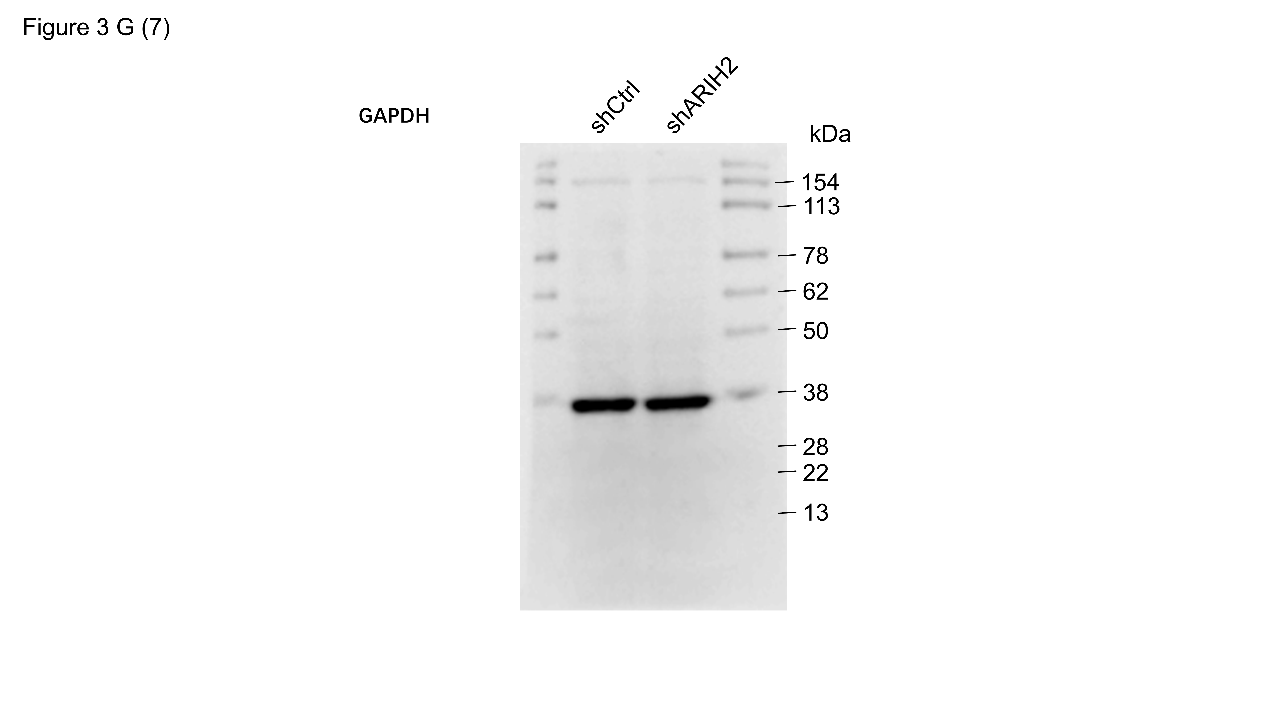

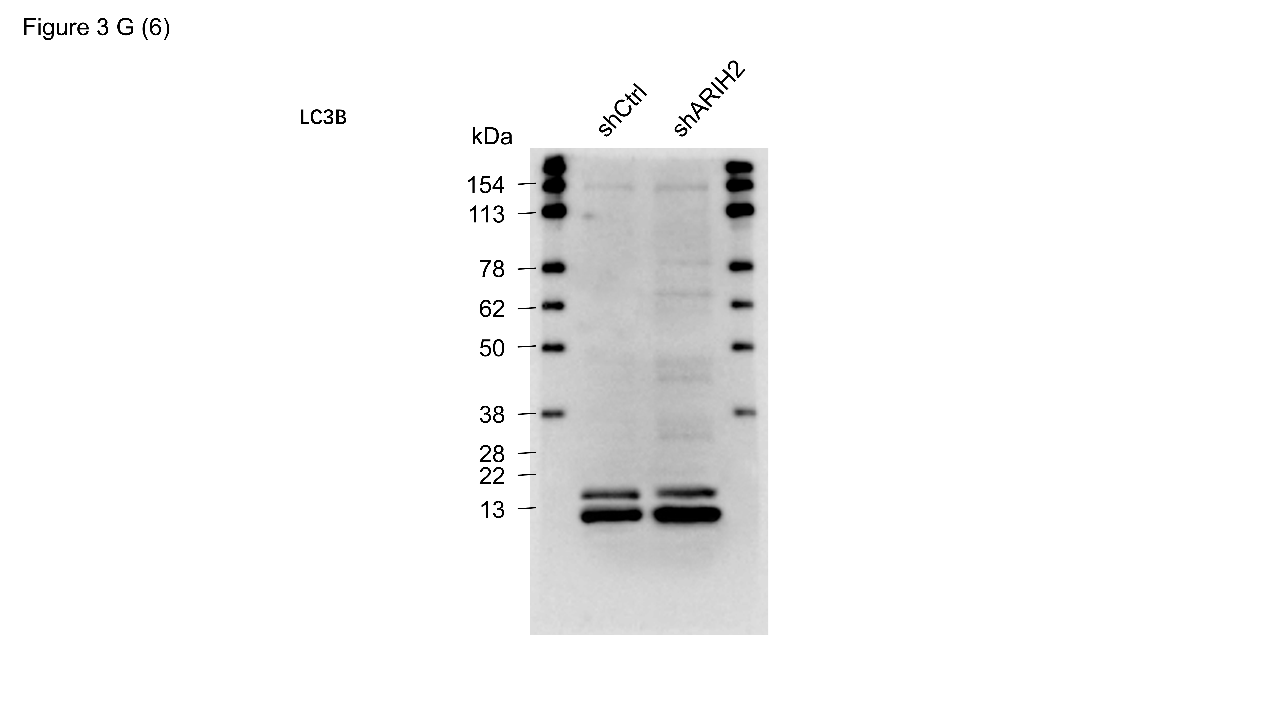

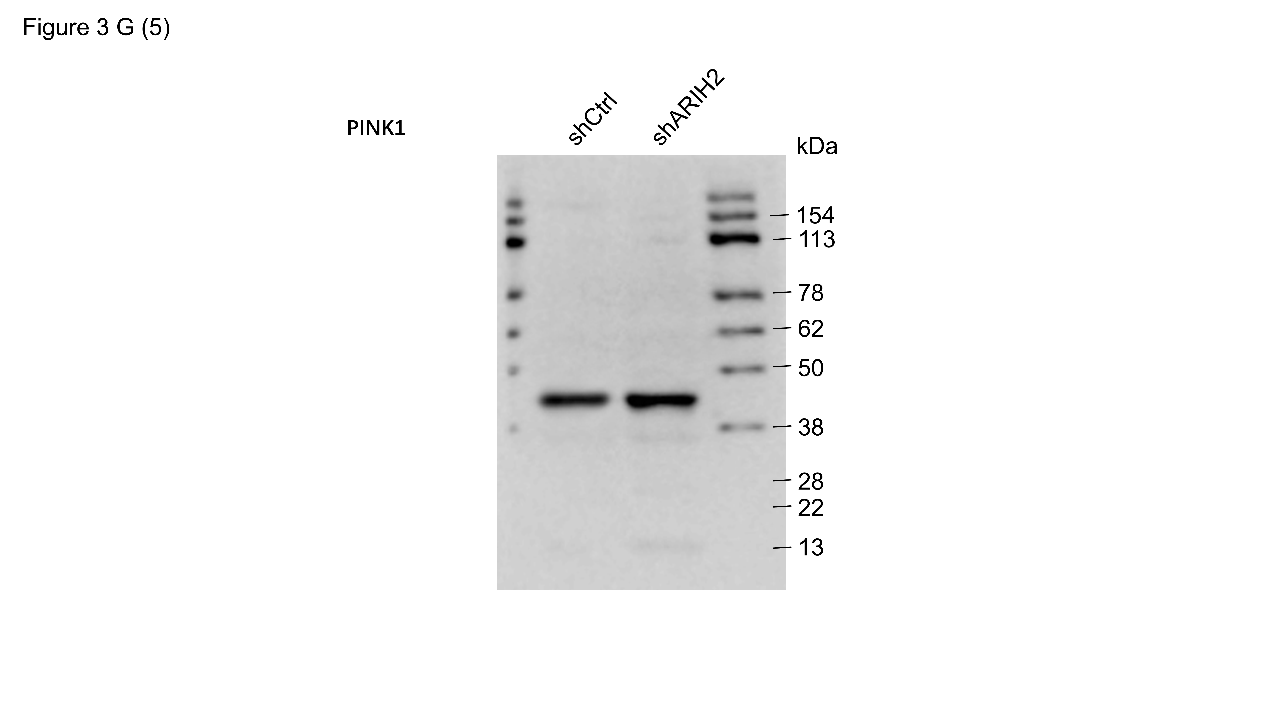
Figure4B PINK1 Figure4B LC3B Figure4B GAPDH

GAPDH

LC3B

PINK1


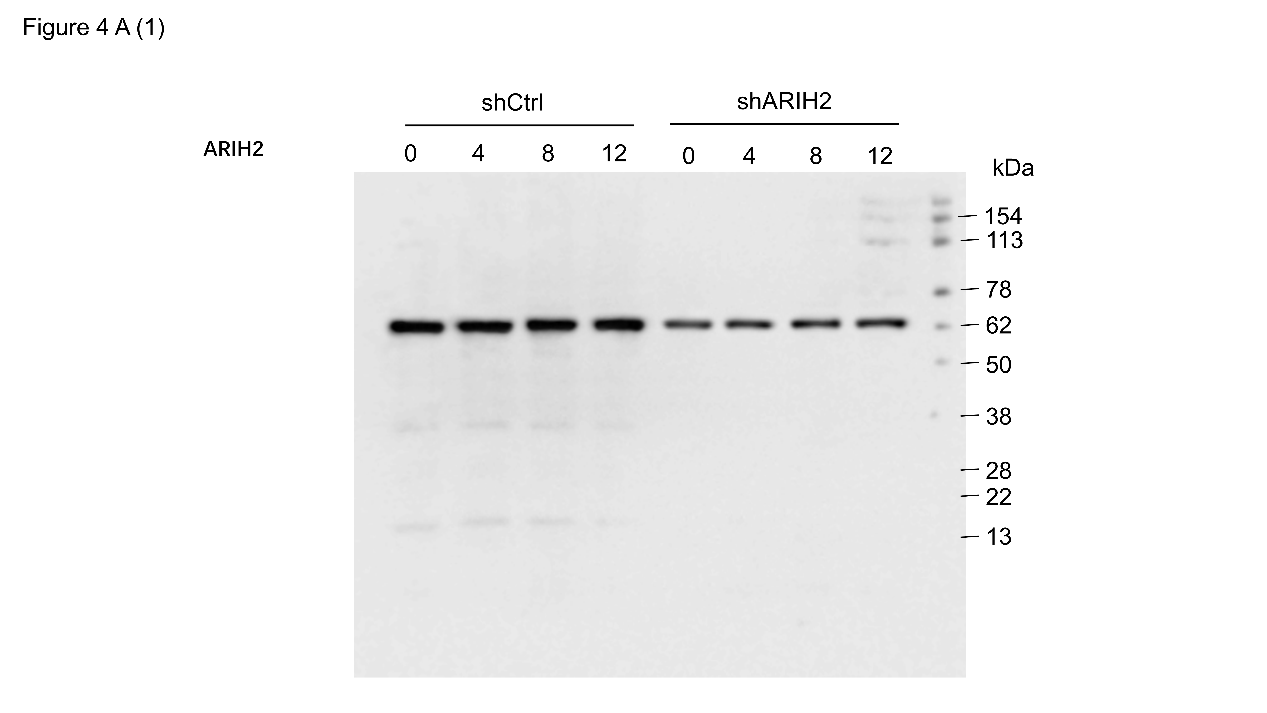
Figure4C ARIH2

ARIH2


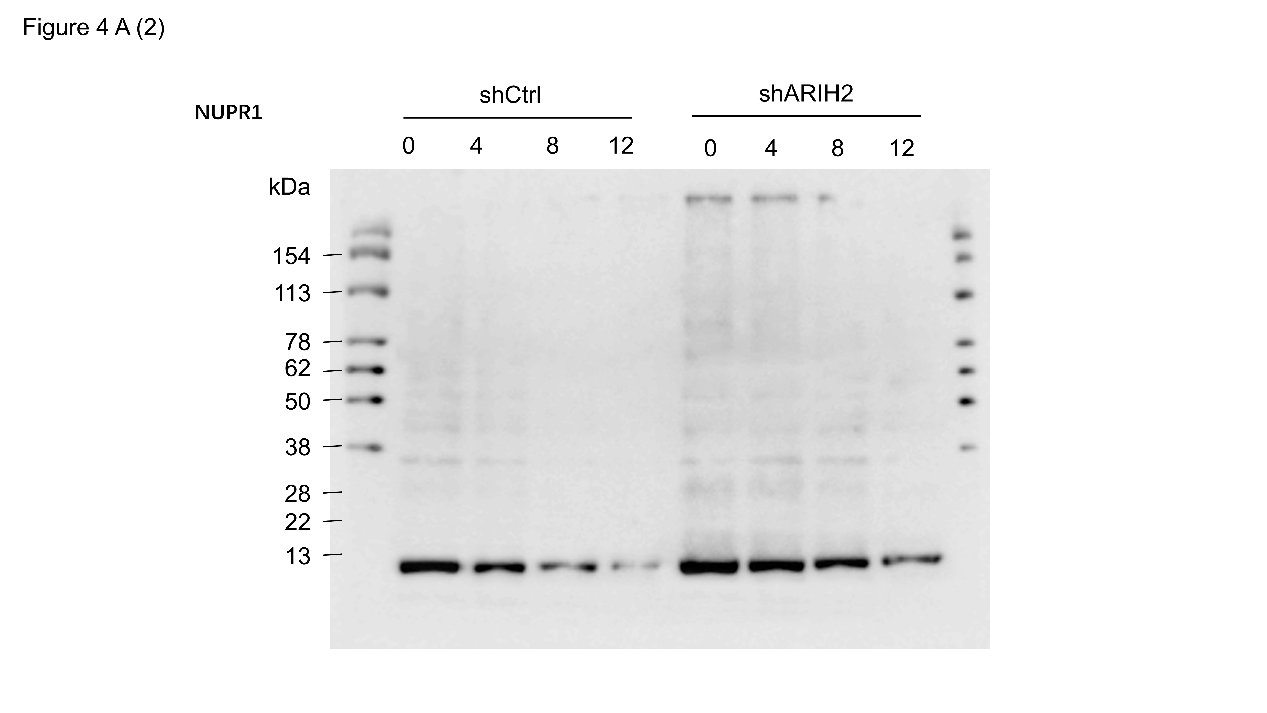
Figure4C NUPR1

NUPR1


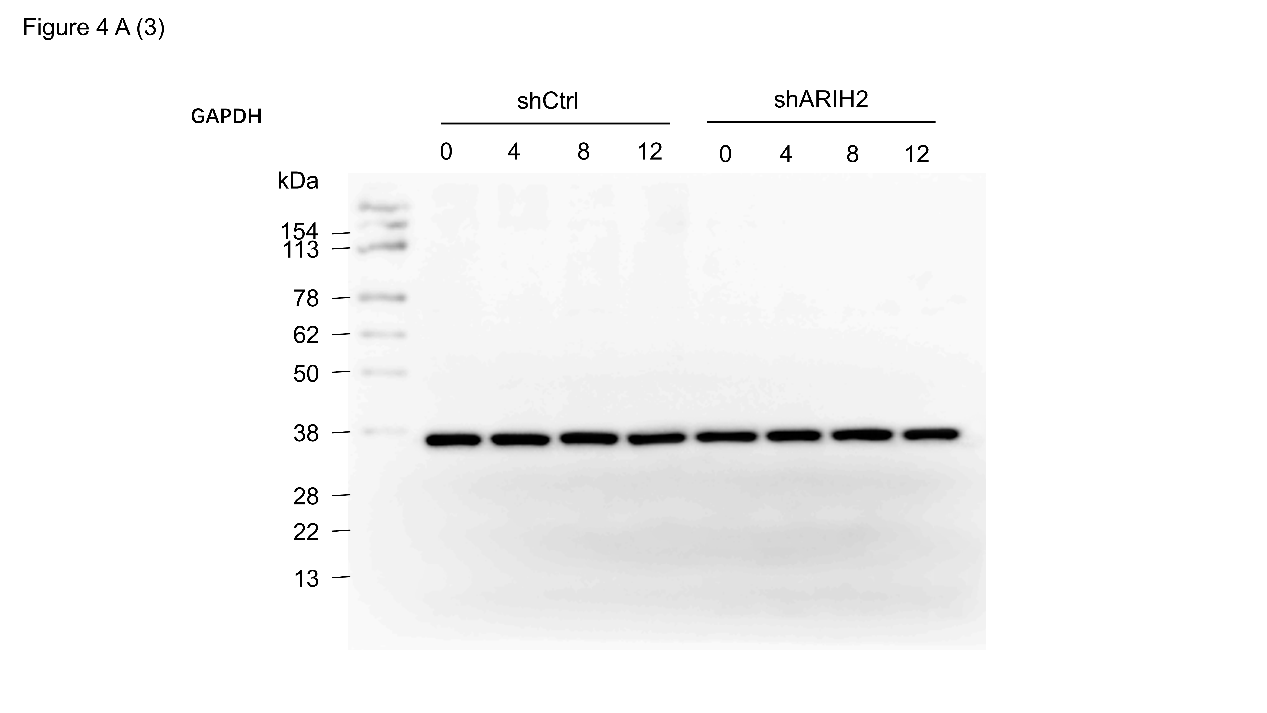
Figure4C GAPDH

GAPDH


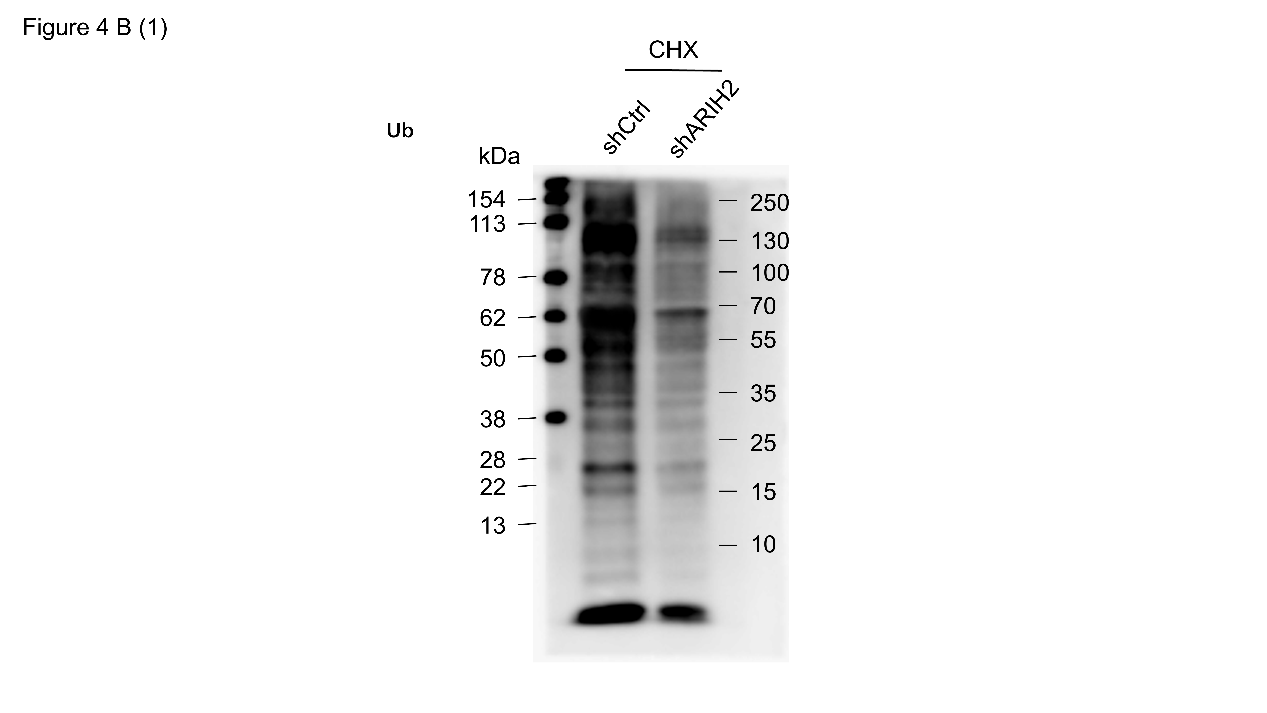

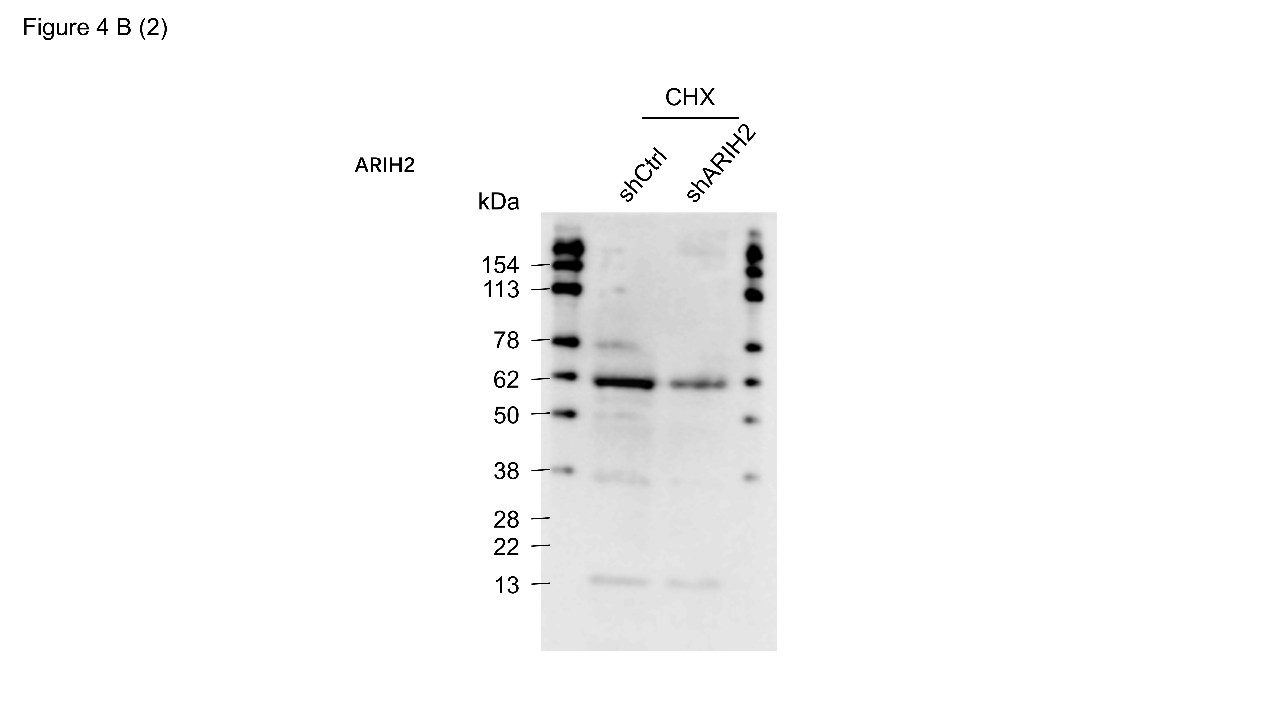
Figure4D IP Ub Figure4D IP ARIH2

ARIH2


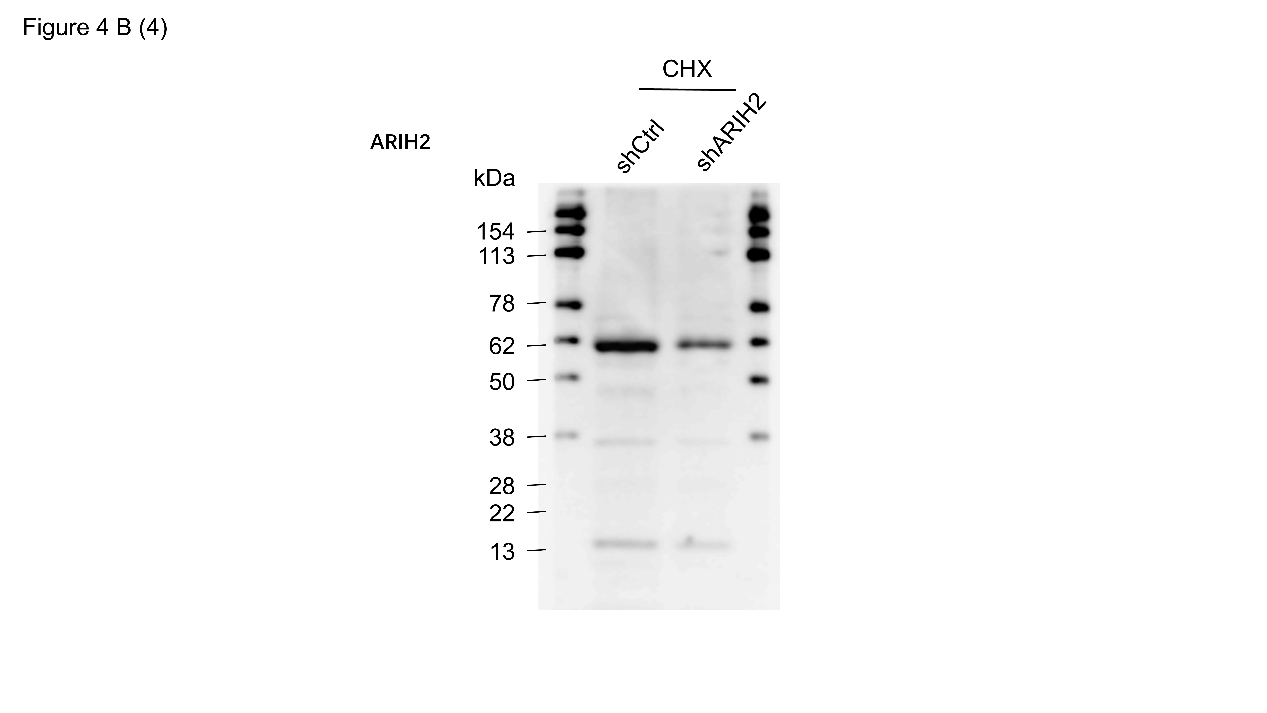

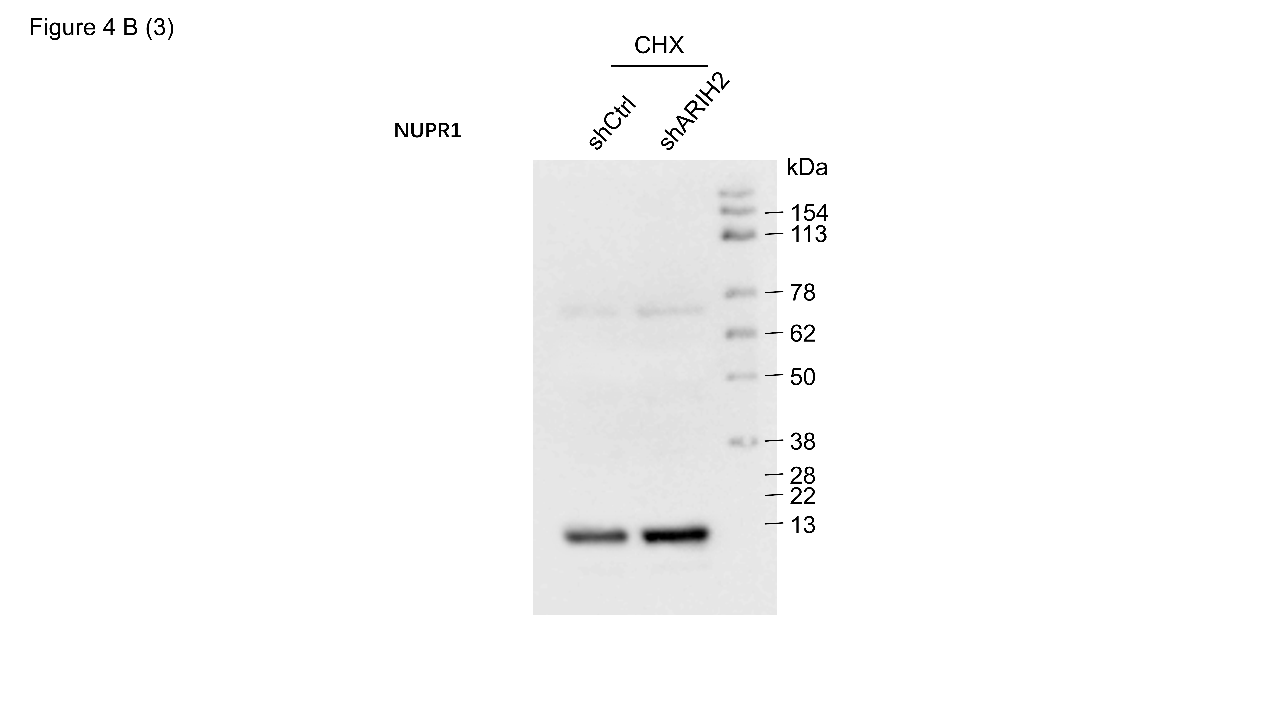
Figure4D IP NUPR1 Figure4D Input ARIH2

ARIH2

NUPR1


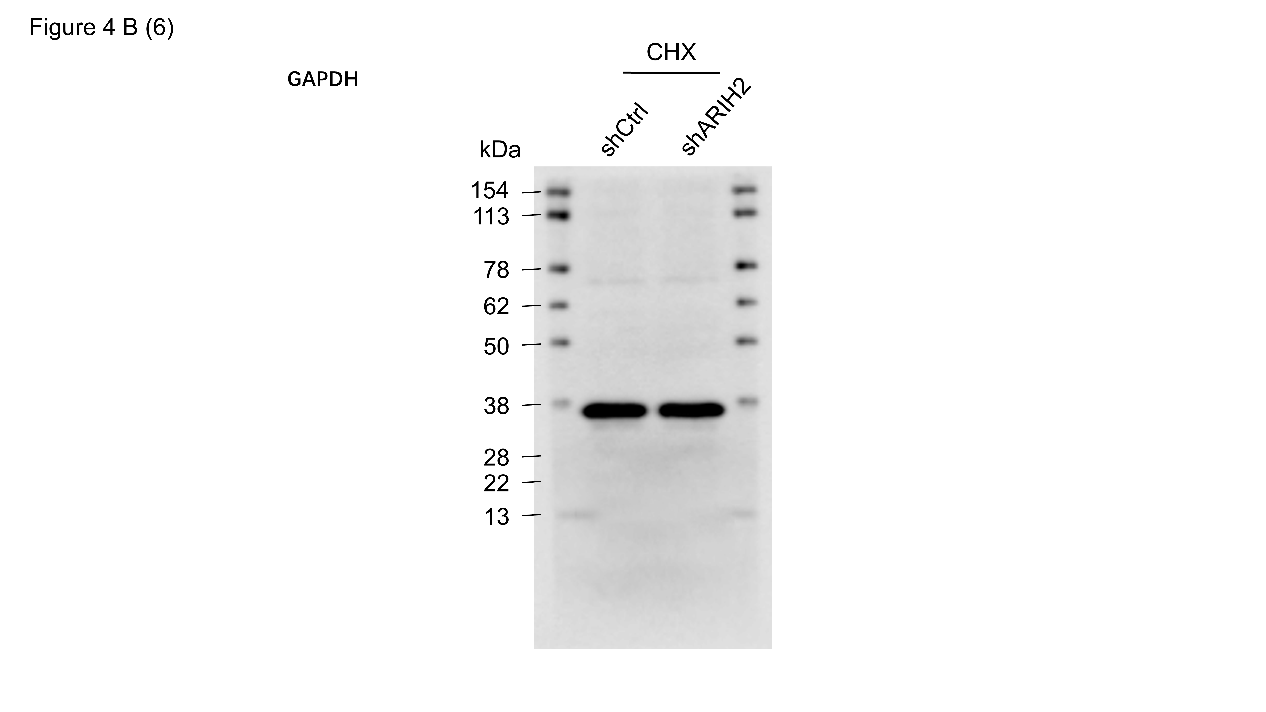

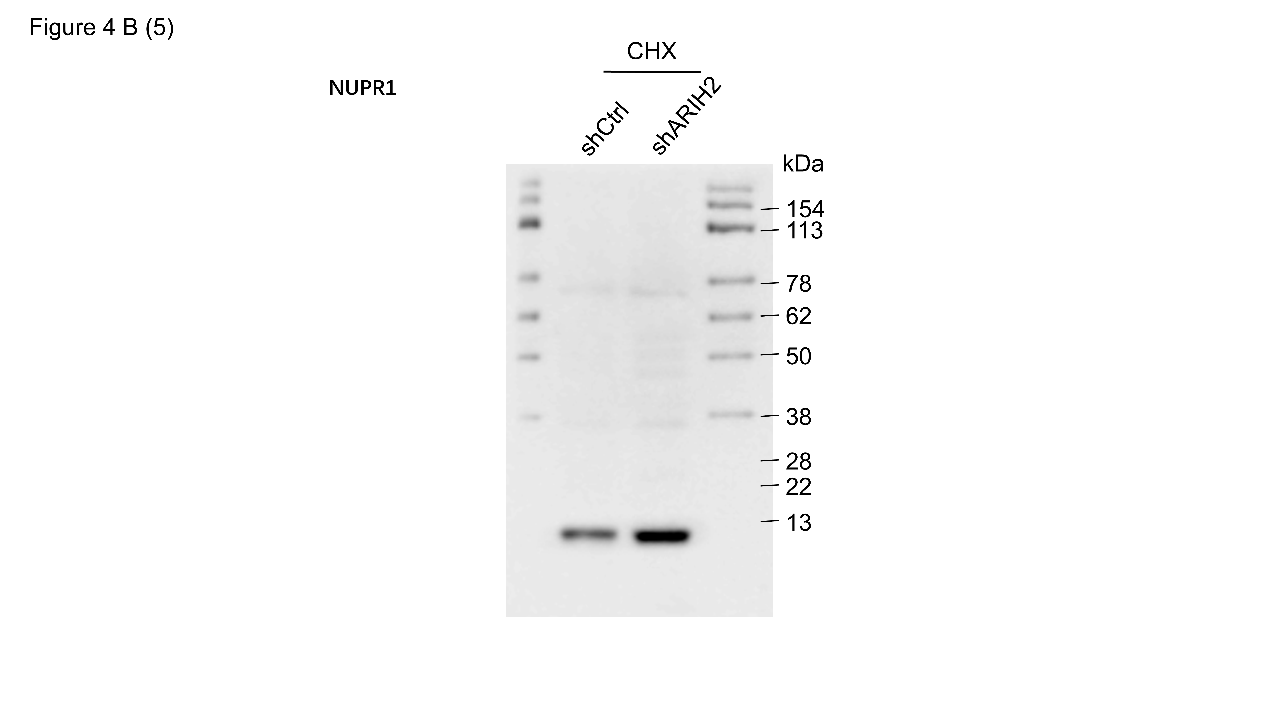
Figure4D Input NUPR1 Figure4D Input GAPDH

GAPDH

NUPR1


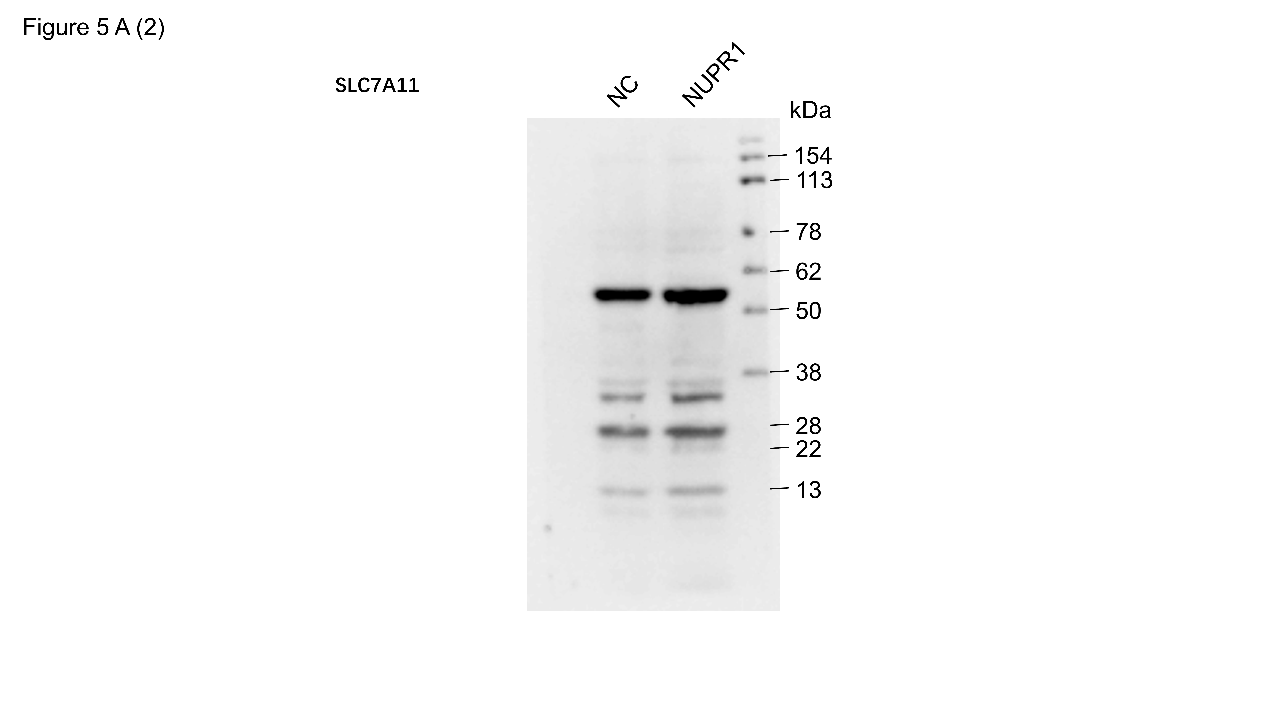

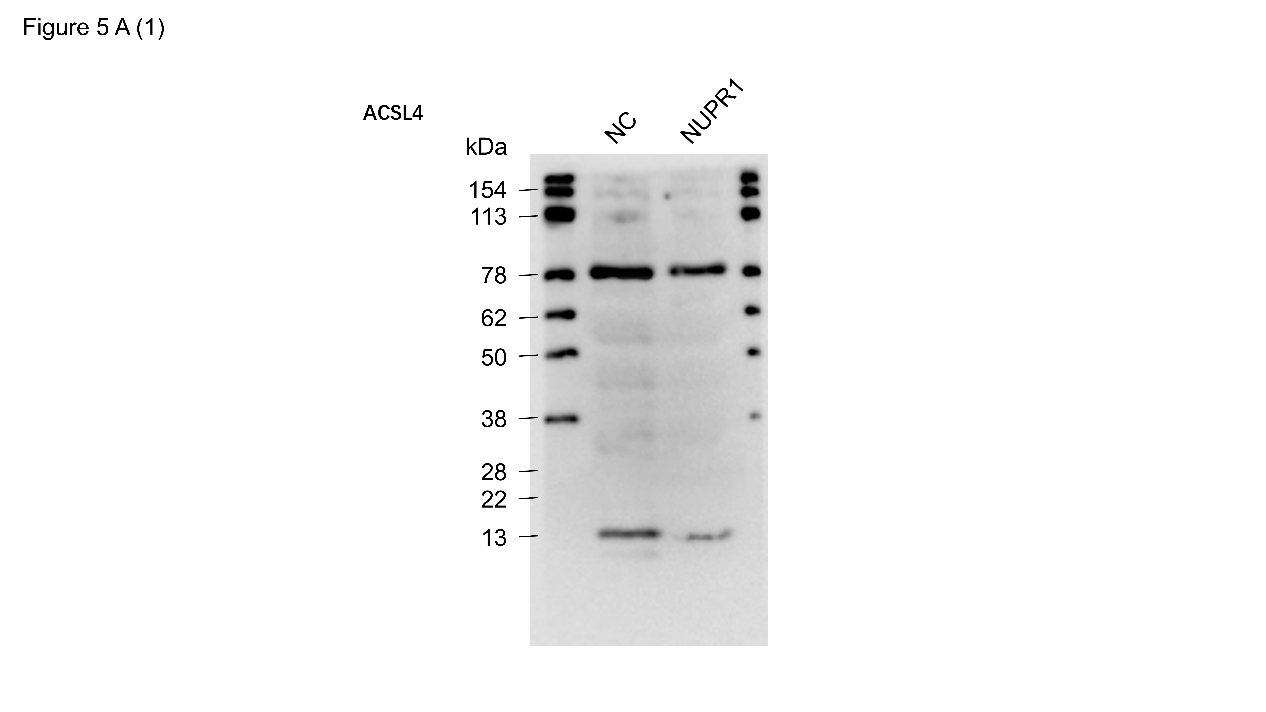
Figure5A ACSL4 Figure5A SLC7A11

ACSL4

SLC7A11


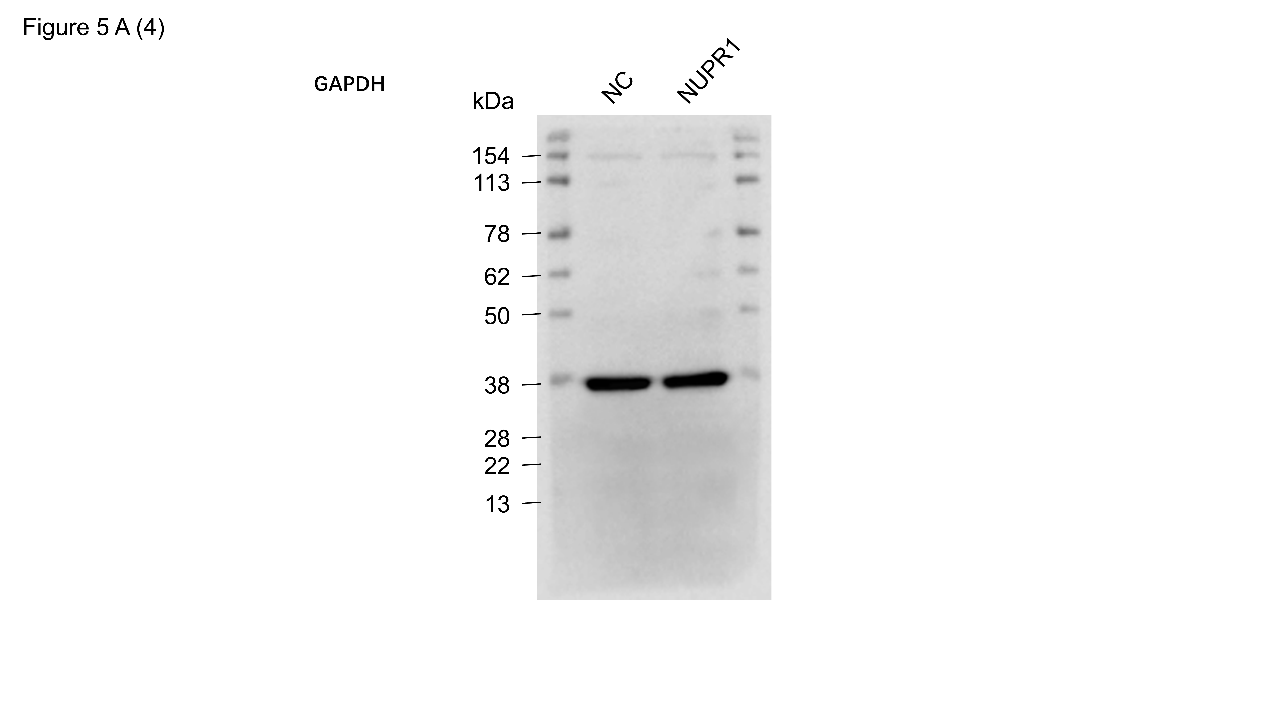

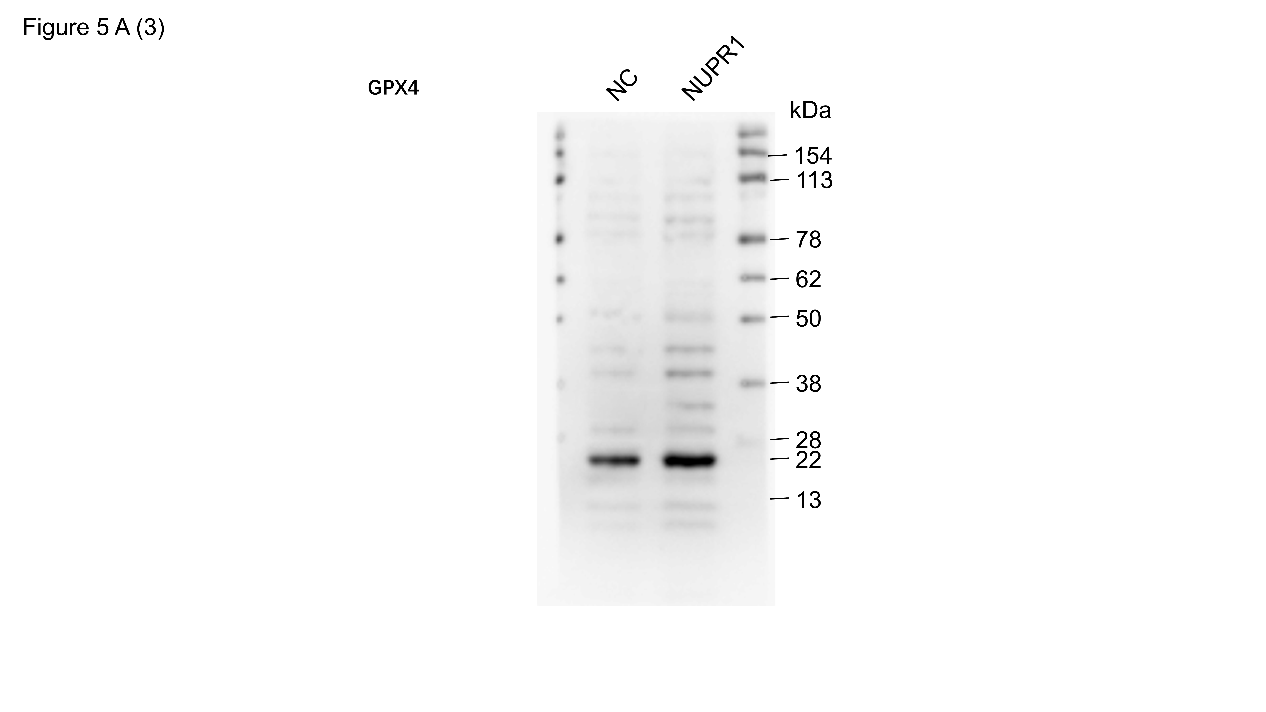
Figure5A GPX4 Figure5A GAPDH

GAPDH

GPX4


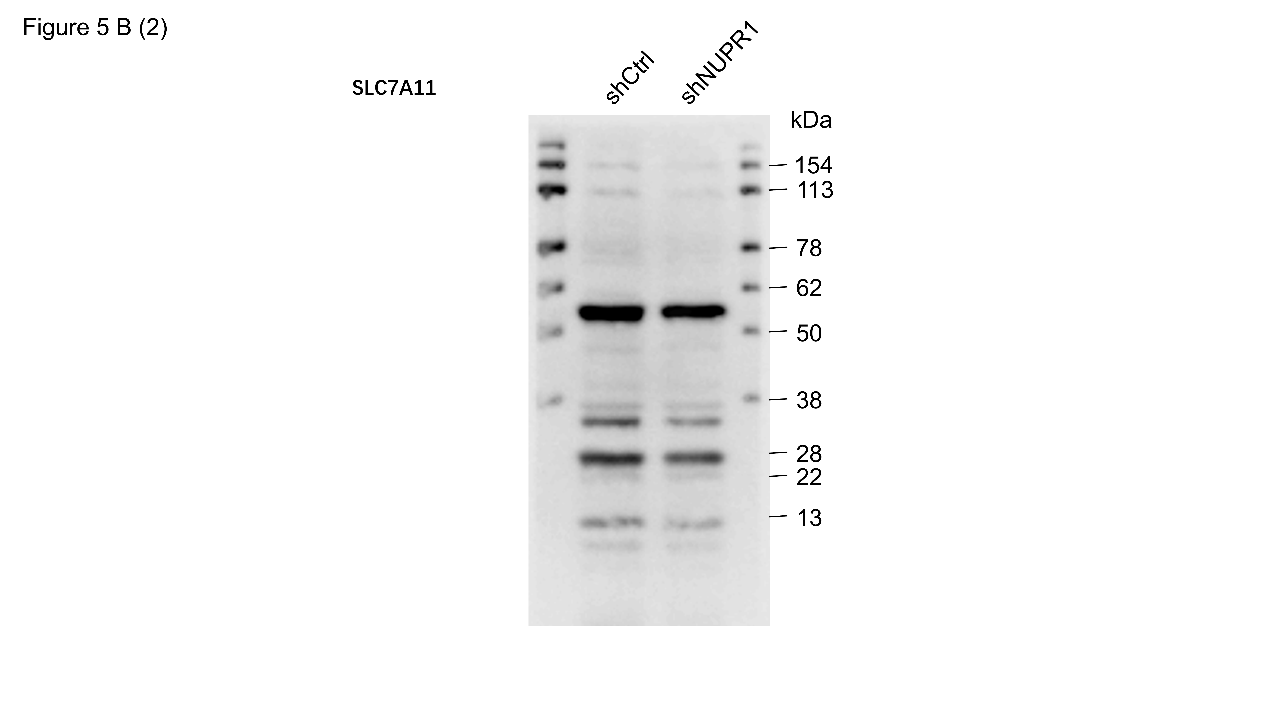

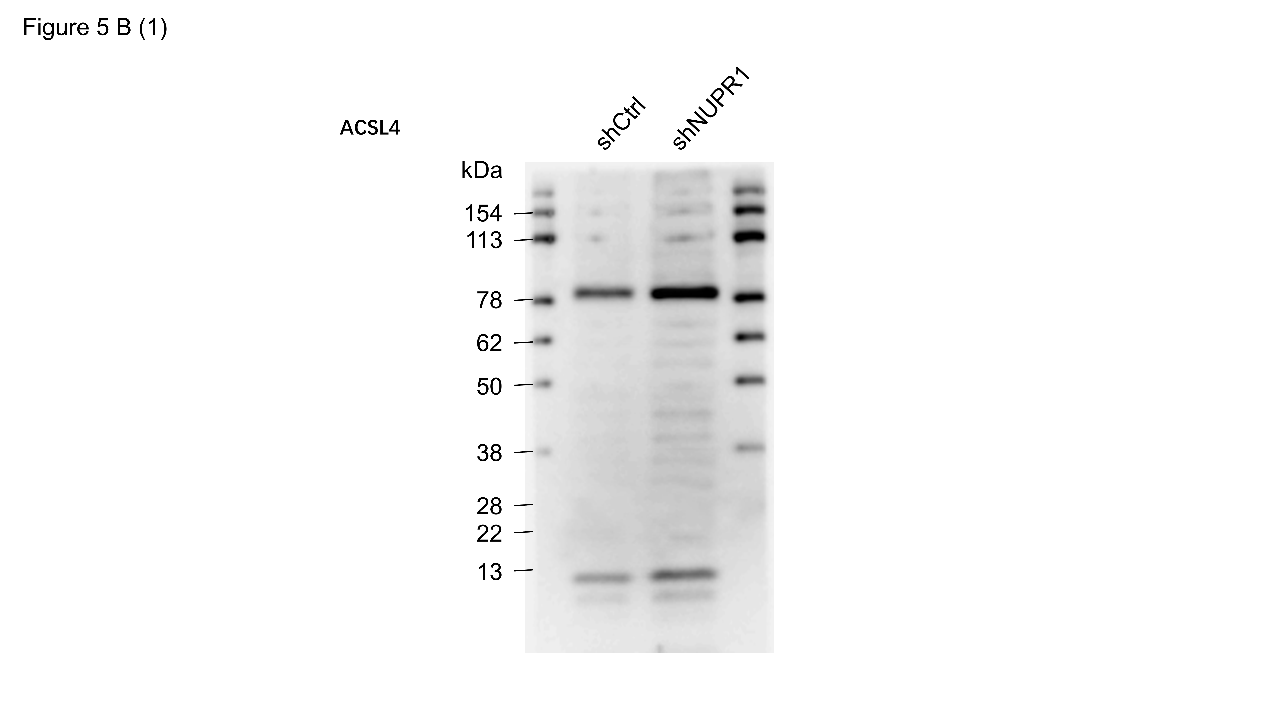
Figure5B ACSL4 Figure5B SLC7A11

SLC7A11

ACSL4


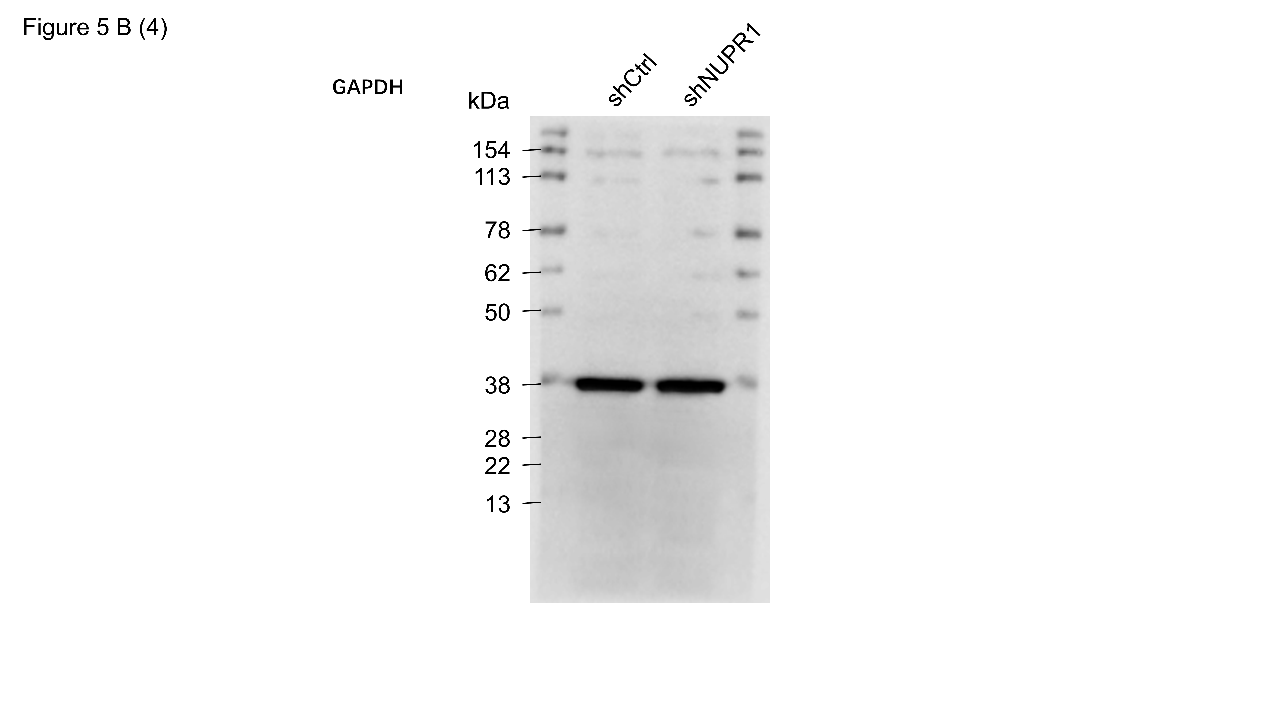

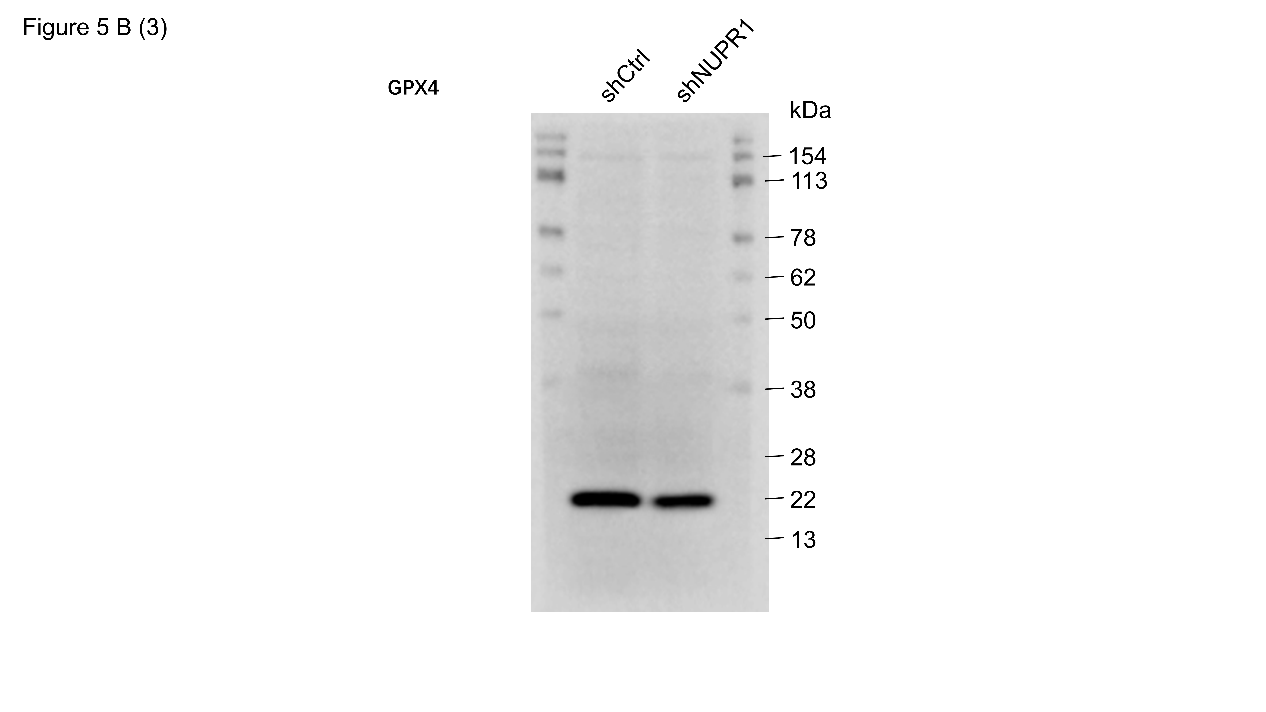
Figure5B GPX4 Figure5B GAPDH

GAPDH

GPX4


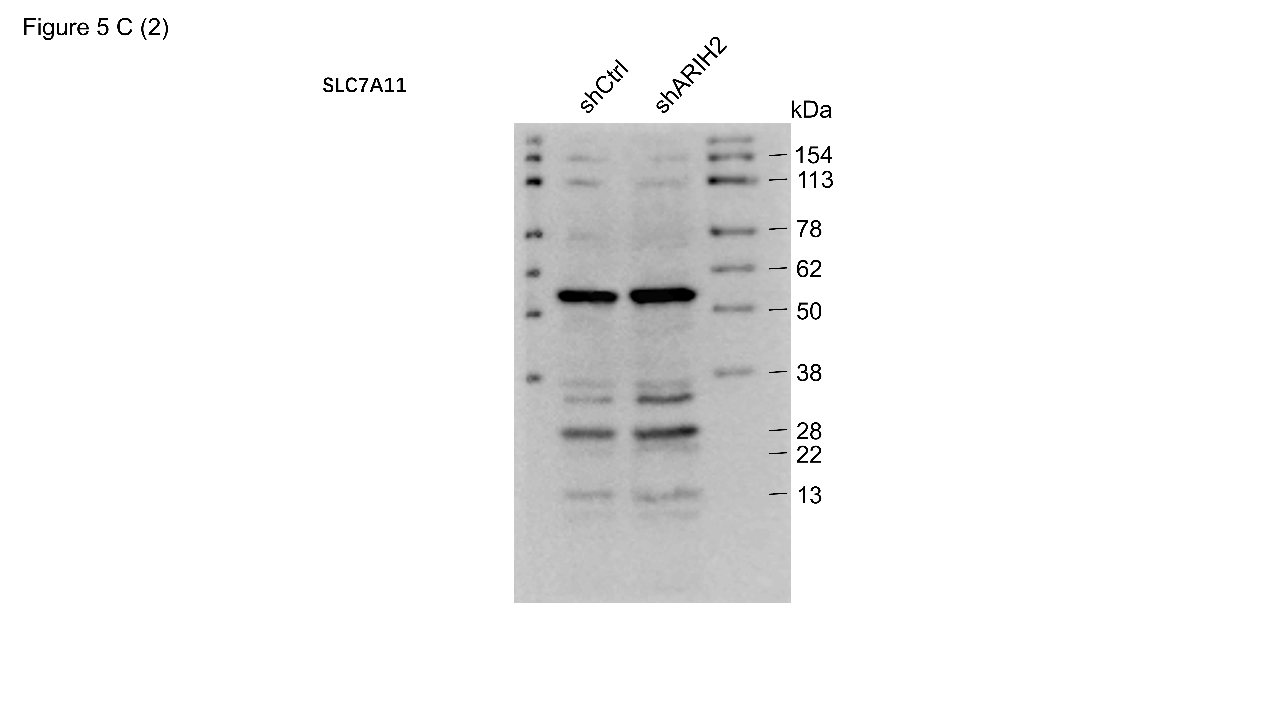

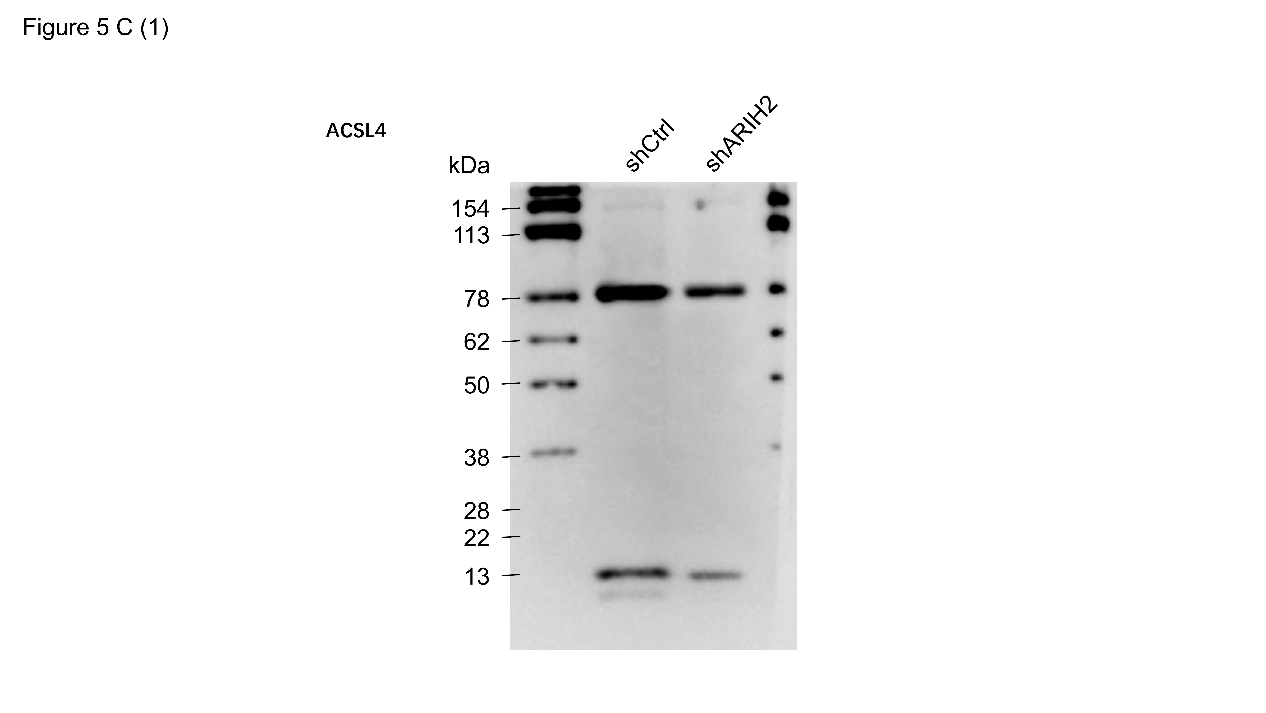
Figure5C ACSL4 Figure5C SCL7A11

ACSL4

SCL7A11


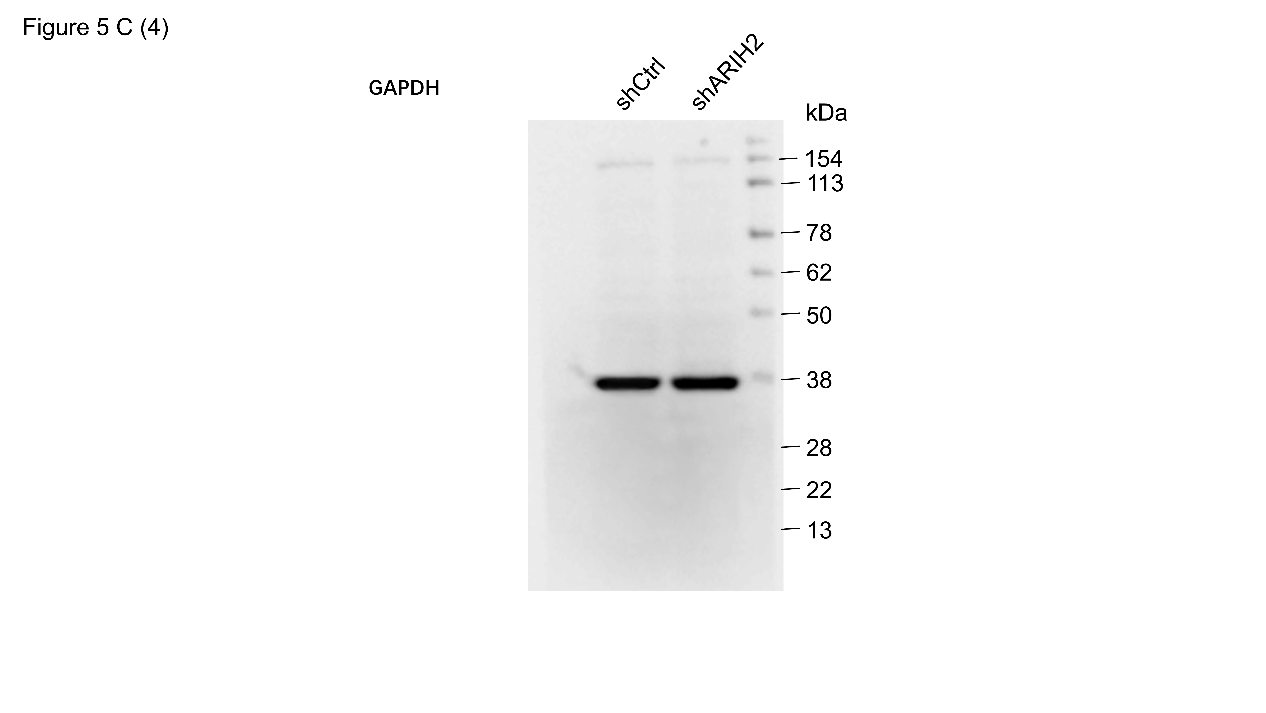

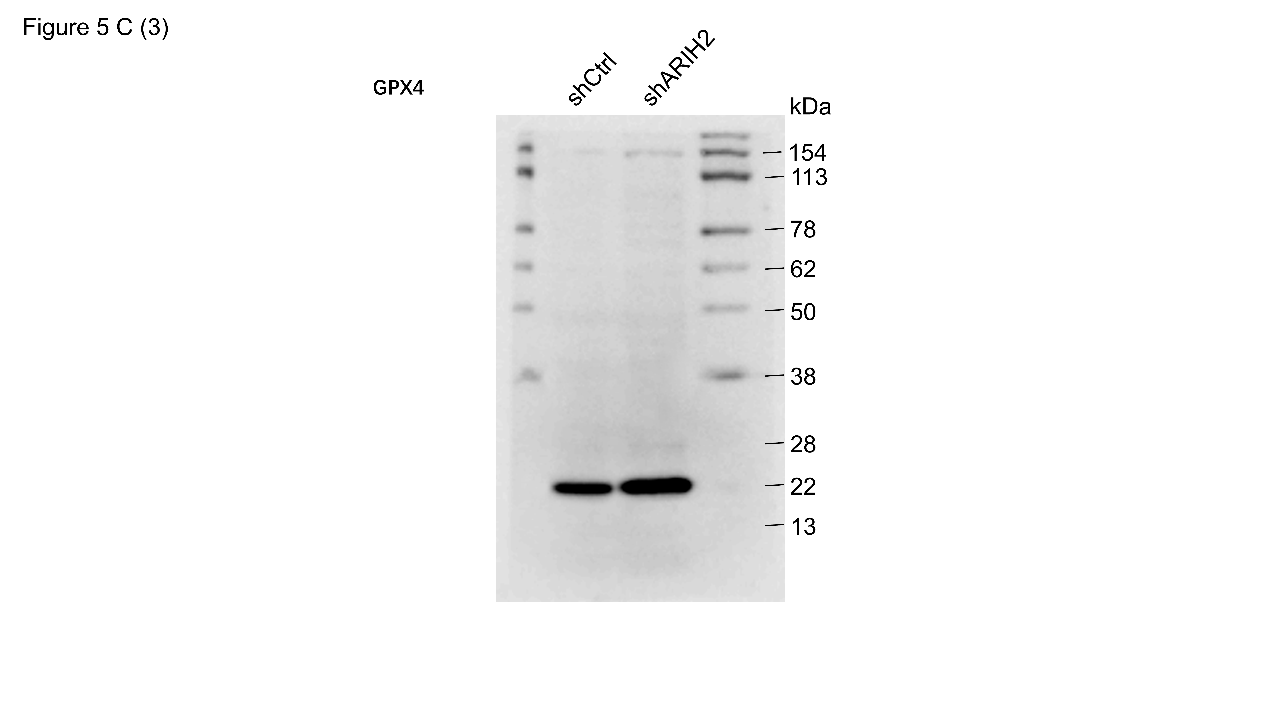
Figure5C GPX4 Figure5C GAPDH

GAPDH

GPX4
